# Regulation of actions and habits by ventral hippocampal trkB and adolescent corticosteroid exposure

**DOI:** 10.1371/journal.pbio.2003000

**Published:** 2017-11-29

**Authors:** Elizabeth T. Barfield, Kyle J. Gerber, Kelsey S. Zimmermann, Kerry J. Ressler, Ryan G. Parsons, Shannon L. Gourley

**Affiliations:** 1 Department of Pediatrics, Emory University, Atlanta, Georgia, United States of America; 2 Graduate Program in Neuroscience, Emory University, Atlanta, Georgia, United States of America; 3 Yerkes National Primate Research Center, Emory University, Atlanta, Georgia, United States of America; 4 Department of Psychiatry and Behavioral Sciences, Emory University, Atlanta, Georgia, United States of America; 5 Graduate Program in Molecular and Systems Pharmacology, Emory University, Atlanta, Georgia, United States of America; 6 Department of Psychology, Graduate Program in Integrative Neuroscience and Program in Neuroscience, Stony Brook University, Stony Brook, New York, United States of America; Institute of Science and Technology Austria, Austria

## Abstract

In humans and rodents, stress promotes habit-based behaviors that can interfere with action–outcome decision-making. Further, developmental stressor exposure confers long-term habit biases across rodent–primate species. Despite these homologies, mechanisms remain unclear. We first report that exposure to the primary glucocorticoid corticosterone (CORT) in adolescent mice recapitulates multiple neurobehavioral consequences of stressor exposure, including long-lasting biases towards habit-based responding in a food-reinforced operant conditioning task. In both adolescents and adults, CORT also caused a shift in the balance between full-length tyrosine kinase receptor B (trkB) and a truncated form of this neurotrophin receptor, favoring the inactive form throughout multiple corticolimbic brain regions. In adolescents, phosphorylation of the trkB substrate extracellular signal-regulated kinase 42/44 (ERK42/44) in the ventral hippocampus was also diminished, a long-term effect that persisted for at least 12 wk. Administration of the trkB agonist 7,8-dihydroxyflavone (7,8-DHF) during adolescence at doses that stimulated ERK42/44 corrected long-lasting corticosterone-induced behavioral abnormalities. Meanwhile, viral-mediated overexpression of truncated trkB in the ventral hippocampus reduced local ERK42/44 phosphorylation and was sufficient to induce habit-based and depression-like behaviors. Together, our findings indicate that ventral hippocampal trkB is essential to goal-directed action selection, countering habit-based behavior otherwise facilitated by developmental stress hormone exposure. They also reveal an early-life sensitive period during which trkB–ERK42/44 tone determines long-term behavioral outcomes.

## Author summary

Stress can impair the ability of individuals to select actions based on their consequences, promoting instead the acquisition of habits—automatic behaviors that are insensitive to action–outcome relationships. Stress in adolescence can have particularly long-lasting consequences, affecting behavior and mood regulation even in adulthood. Here, we show that adolescent mice exposed to a stress hormone developed biases towards habits and amotivation (a symptom of depression) in adulthood. We investigated the role of the stress-sensitive tyrosine kinase receptor B (trkB) receptor, a protein that regulates synaptic strength and plasticity in the mammalian nervous system. This receptor was modified by adolescent experience throughout multiple brain regions, but the changes were particularly long lasting in the ventral hippocampus—a brain region involved in mood regulation. Experimentally reducing the activity of the receptor recapitulated the behavioral abnormalities induced by the stress hormone. Meanwhile, pharmacological stimulation of the receptor corrected these abnormalities and had long-lasting antidepressant-like effects that persisted even after drug treatment ended. Thus, pharmacological interventions that augment trkB activity may be particularly efficacious in treating neurobehavioral deficits in adolescents exposed to chronic stress.

## Introduction

Goal-directed actions are defined as behaviors directed towards achieving a specific outcome. By contrast, habits are stimulus elicited and insensitive to action–outcome relationships. Individuals who experience early-life stress have an increased incidence of behaviors that can lead to addiction and obesity as adults, and Patterson et al. [1] provided evidence that these behaviors may result from an overreliance on outcome-insensitive habits. In rats, chronic stressor exposure similarly biases behavioral response strategies towards habits [2], and the primary glucocorticoid corticosterone (CORT) is sufficient to induce habit biases in both rats and mice [3]. Exogenous glucocorticoids similarly enhance habit-based learning and memory in humans [4]. And like early-life stress [1], prenatal stress in humans and maternal separation in neonatal rats also induce inflexible habit behavior [5,6].

Despite these convergences across species, how elevated glucocorticoids, particularly during specific developmental periods, cause long-term biases towards habit-based behavior remains unclear. To address this issue, we elevated CORT in mice during a timespan equivalent to early adolescence in humans, which induced habit biases in adulthood. We hypothesized that adolescent CORT exposure may have long-term behavioral consequences by impacting tyrosine kinase receptor B(trkB), the high-affinity receptor for Brain-derived Neurotrophic Factor (BDNF), in corticolimbic regions. We were motivated by evidence that corticohippocampal trkB levels increase during early postnatal development and adolescence [7] and are stress sensitive [8].

Our investigations focused on the ventral hippocampus (vHC), medial prefrontal cortex (mPFC), striatum, and amygdala, brain regions implicated in action–outcome decision-making—that is, in selecting behaviors based on expected consequences, rather than familiar habit-based strategies [9–13]. Specifically, inactivation of the mPFC or connected regions of the striatum causes failures in selecting actions based on their outcomes or on outcome value [9–13]. Similar behavioral impairments follow amygdala inactivation (in particular, inactivation of the basolateral compartment [13]). Meanwhile, vHC inactivation disrupts goal encoding in the mPFC [14,15], and the vHC appears to route contextual and task-relevant information to the mPFC and amygdala in order to influence reward-related decision-making and response selection [16,17].

Habit biases that occur due to disruptions in corticolimbic networks may be associated with depression-like behavior. Depressive rumination in humans can be habit-like—stimulus elicited, resistant to change, and precipitated by stressor exposure [18]. A sense of helplessness in depression has also been conceptualized as a habit-based weakness in awareness of action–outcome contingency [19]. To investigate habit biases following adolescent CORT exposure, we used an instrumental contingency degradation procedure in which a familiar behavior was uncoupled from reward. To investigate depression-like behavior, we turned to the progressive ratio task, a classical assay of reward-related motivation. Using these separable strategies, our findings suggest that vHC trkB is necessary for goal-directed action (occluding habits) and that compromised trkB signaling induces habit-based and depression-like behavior. They also reveal a sensitive period during which enhancing ERK42/44 activity during adolescence can interfere with CORT-induced habit-based and depression-like behavior later in life.

## Results

### Exogenous CORT exposure recapitulates several effects of stress: Validation of the method

In both humans and rodents, glucocorticoid exposure can induce biases towards habit-based behavior, at the expense of goal-directed action [3,4]. Furthermore, stressor exposure during early developmental periods appears to confer long-term habit biases across rodent–primate species [1,5,6]. Despite these homologies, mechanisms remain unclear. To address these issues, we exposed mice to CORT in the drinking water from postnatal day (P) 31–42, equivalent to early adolescence in humans [20]. The timing of experimental events is provided in Table 1, and timelines are also provided in the figures.

**Table 1:** List of experiments. A summary of experiments reported in this manuscript is provided, with corresponding figure numbers. “Adult” refers to testing completed between 8–12 wk of age.

Group	Figure	Age of CORT/stressor exposure	Additional manipulation, if any	Age of testing	End points
1	1a,b	CORT: P31–42	n/a	P42 versus P125	Blood serum CORT and glands
2	1c	CORT: P31–42	n/a	adult	Progressive ratio test
3	1d	swim stress: P31–42	n/a	P31 versus P42	Blood serum CORT
4	1e	swim stress: P31–42	n/a	adult	Progressive ratio test
5	1f-h, S1 Fig	CORT: P31–42	n/a	P42 versus P125	Dendritic spine analysis
6	1i	CORT: P31–42	Acute stressor	adult	Forced swim test
7	2a,b,f, S3 Table	CORT: P31–42	n/a	P56	Instrumental contingency degradation, reinforcer devaluation
8	2c	CORT: P31–42	n/a	P56	Instrumental contingency degradation with context shift
9	2d-f, S3 Table	CORT: P56–67/68	n/a	P82	Instrumental contingency degradation, reinforcer devaluation
10 F	S2 Fig	CORT: P31–42	n/a	P56	Instrumental contingency degradation
11	2g,h	CORT: P31–42	n/a	P86	Instrumental contingency degradation
12	3a-d, Table 3	CORT: P31–42	n/a	P42	Western blotting
13	3e	CORT: P31–42	n/a	P125	Western blotting
14	3f,g	CORT: P56-67	n/a	P67	Western blotting
15	4a-c	n/a	7,8-DHF	n/a	Western blotting
16	4d-g, S2 Table	CORT: P31–42	7,8-DHF	adult	Instrumental contingency degradation, followed by forced swim test, followed by locomotor monitoring
17	4h	CORT: P31–42	7,8-DHF	adult	Progressive ratio test
18 F	S2 Fig	CORT: P31–42	7,8-DHF	adult	Instrumental contingency degradation
19	Table 4, S1 Table	CORT: P31–42	7,8-DHF	P125	Western blotting, glands
20	5a-d, S3 Fig	n/a	*Trkb*.*t1* virus	adult	Instrumental contingency degradation, immunostaining
21	5e-g, S3 Fig	n/a	*Trkb*.*t1* virus	adult	Repeated instrumental contingency degradation, followed by progressive ratio

**Abbreviations**: 7,8-DHF, 7,8-dihydroxyflavone; CORT, corticosterone; F, female mice; P, postnatal day; Trkb.t1, truncated trkB

**Fig 1:**
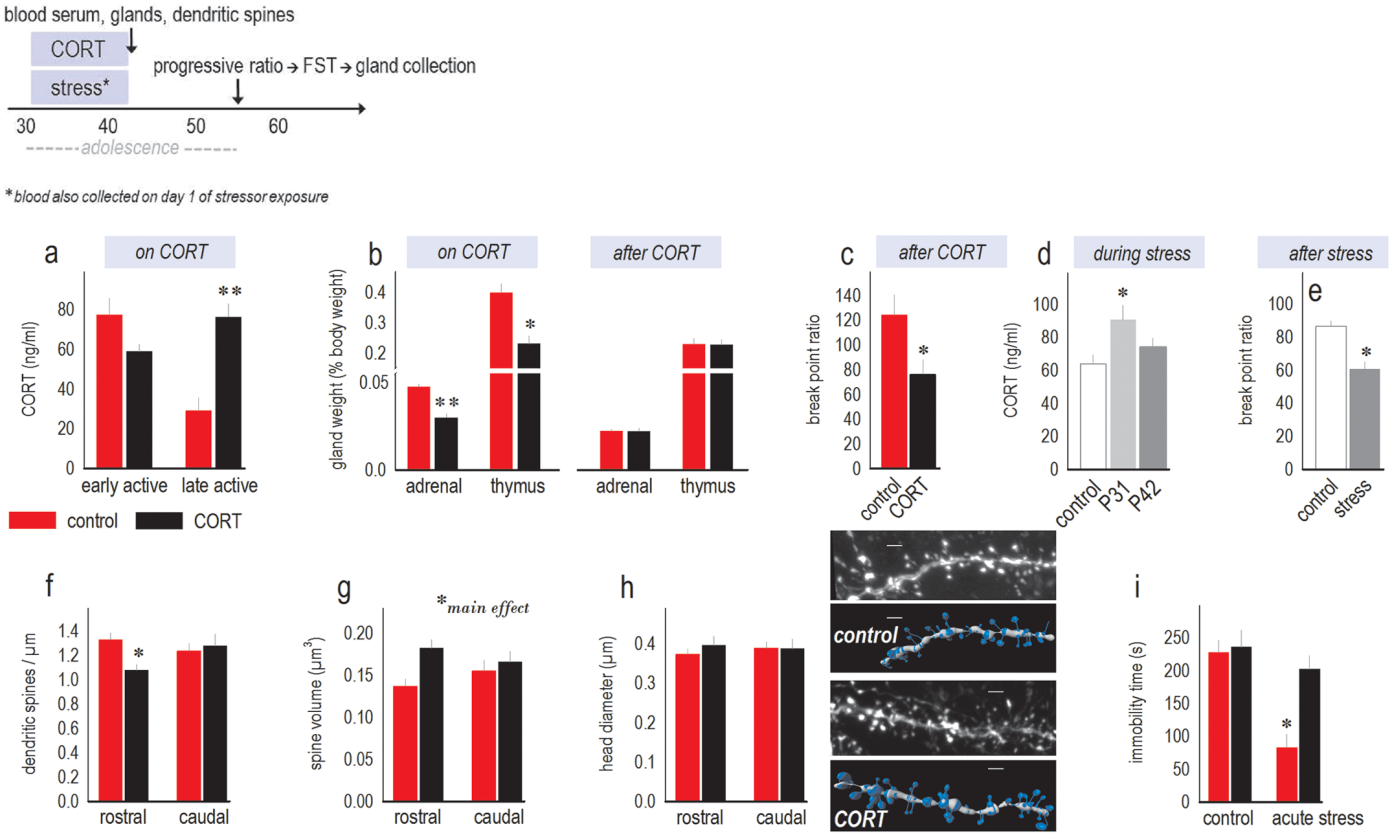
Effects of CORT exposure during adolescence: Validation of the procedure. Top: timeline of experimental events. See also Table 1. (a) Blood serum CORT levels at the end of an 11-d CORT exposure period (from P31–42) did not differ between groups at the beginning of the active cycle (following sleep) but were elevated in CORT-drinking mice (relative to control mice) at the end of the awake, active cycle. *n* = 5–10/group. (b) Adrenal and thymus gland weights also atrophied following exogenous CORT exposure (left), but glands recovered with a washout period (right). *n* = 5–6/group. (c) In a progressive ratio test, a history of CORT exposure reduced break point ratios. *n* = 5–6/group. (d) Forced swim stress in adolescence also increased blood serum CORT (though this effect appeared to habituate with repeated exposure). *n* = 5–11/group. (e) Further, break point ratios were also reduced, as with CORT exposure. *n* = 5–10/group. (f) CORT exposure from P31–42 also eliminated dendritic spines on excitatory neurons in the anterior mPFC (prelimbic subregion) of *thy1*-YFP expressing transgenic mice. *n* = 7 mice/group. (g) CORT increased overall spine volume. (h) Dendritic spine head diameter did not, however, change. Representative dendrites are adjacent (unprocessed image at top, followed by reconstruction). (i) Finally, in the forced swim test, a history of subchronic CORT exposure did not impact mobility but modified reactivity to an acute stressor: Acute stress induced mobility in control mice, but mice with a history of CORT exposure remained immobile. *n* = 5–7/group. * *p* ≤ 0.05, ** *p* < 0.001 compared to control (red or white bars). Scale bar = 2 μm. Raw data for this figure can be found in S1 Data. CORT, corticosterone; FST, forced swim test; mPFC, medial prefrontal cortex; P, postnatal day; YFP, yellow fluorescent protein.

We first confirmed that exogenous CORT exposure elevated blood serum CORT late in the active period (that is, nighttime) when mice had been active and ingesting CORT for several h. Notably, levels did not differ between CORT-exposed and control groups during the early active period when mice were just waking (interaction *F*_(1,27)_ = 22.3, *p* < 0.001) (Fig 1a). This pattern indicates that exogenous CORT disrupts typical diurnal changes in blood serum CORT levels. Correspondingly, adrenal and thymus glands atrophied during the CORT exposure period, as expected (*t*_8_ = 5.7, *p* < 0.001; *t*_8_ = 4.24, *p* = 0.003) (Fig 1b). Also as expected, gland weights recovered when exogenous CORT was removed (*t*_10_ = −0.25, *p* = 0.8; *t*_10_ = 0.099, *p* = 0.9) (Fig 1b). Despite this recovery, break point ratios in a progressive ratio test, an assay of reward-related motivation, were reduced in mice with a history of CORT exposure (*t*_9_ = 2.39, *p* = 0.04) (Fig 1c). This pattern is consistent with amotivation in depression and provides evidence of long-term behavioral consequences of adolescent CORT exposure.

We next compared our oral CORT exposure procedure to daily forced swim stress. Forced swimming increased blood serum CORT as expected, although this effect appeared to habituate with repeated exposure (*F*_(2,20)_ = 4.0, *p* = 0.04) (Fig 1d). Nonetheless, adolescent stressor exposure decreased progressive ratio break points, as with the oral CORT procedure (*t*_13_ = 3.56, *p* = 0.003) (Fig 1e).

Another well-characterized consequence of repeated stressor exposure is the elimination of dendritic spines on pyramidal mPFC neurons [21]. Thus, as another experiment validating our CORT exposure method, we enumerated dendritic spines on excitatory deep-layer pyramidal neurons in the mPFC using *thy1*-yellow fluorescent protein (YFP)–expressing transgenic mice. Spines were eliminated in the anterior-most sections (interaction *F*_(1,73)_ = 4.9, *p* = 0.03) (Fig 1f). The effect size (Cohen’s *d*) was 0.92, signaling that approximately 80% of dendrites in CORT-exposed mice had fewer dendritic spines than the control mean.

Dendritic spines were also reconstructed in 3D, revealing increased volume following CORT (main effect *F*_(1,72)_ = 6.2, *p* = 0.02) (Fig 1g). This phenomenon could not be accounted for by an increase in the head size (*F*s < 1) (Fig 1h), suggesting that CORT induced dysmorphic spines with aberrantly large necks. This pattern was detected even several weeks following CORT exposure (S1 Fig).

Lastly, we exposed adult mice with a history of adolescent CORT treatment to the forced swim test. In this test, attempting to escape has been termed “active coping,” while immobility has been termed “passive coping” [22]. Prior CORT exposure did not impact baseline immobility scores; however, an acute stressor challenge prompted an active coping style in control mice, reducing immobility. CORT-exposed mice were, by comparison, more immobile, favoring a passive response (CORT × stress *F*_(1,21)_ = 6.3, *p* = 0.02) (Fig 1i). Thus, exposure to exogenous CORT in adolescence modified stressor reactivity in adulthood.

### Subchronic CORT exposure in adolescence, but not adulthood, induces habit biases

**Fig 2:**
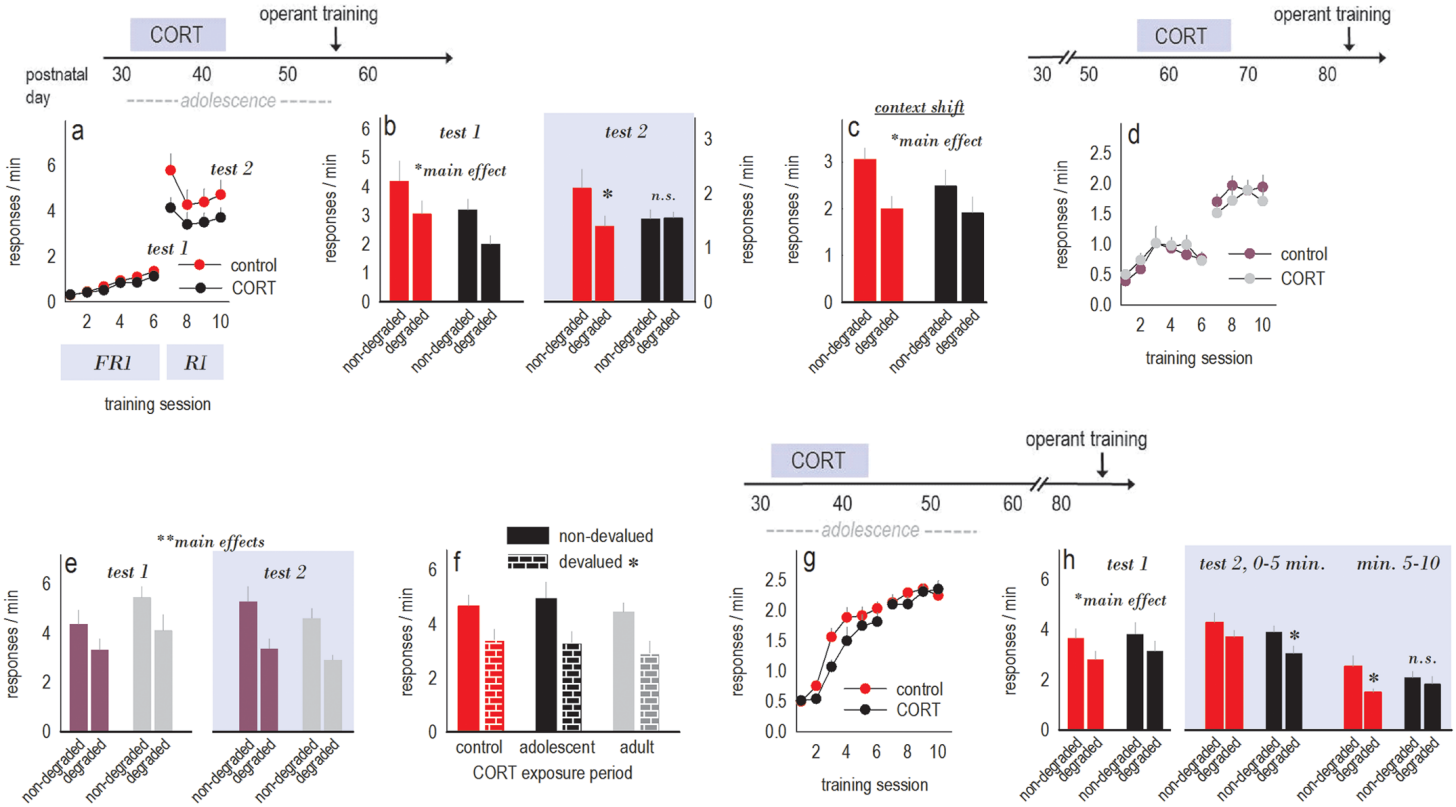
Greater vulnerability to CORT-induced habits in adolescents than adults. Experimental timelines are positioned above the response acquisition curves associated with each experiment. Response acquisition curves represent both responses/min, and breaks in the curves represent tests for sensitivity to instrumental contingency degradation. (a) Instrumental response acquisition was intact 2 wk following adolescent CORT exposure. Text below the *x* axis notes the schedules of reinforcement used throughout (FR1 before test 1, followed by an RI schedule). (b) Sensitivity to instrumental contingency degradation was also initially intact (test 1) in that mice inhibited a response that was unlikely to be reinforced (“degraded” condition), but CORT-exposed mice then developed habit-based response strategies, failing to differentiate between responses (test 2). *n* = 8–9/group. (c) In a separate group, test 2 was conducted in a distinct environment (“context shift”). In this case, all mice preferentially engaged the response most likely to be reinforced in a goal-directed manner, indicating that CORT-induced habits (in b) are context dependent. *n* = 7/group. (d) A history of subchronic CORT exposure in adulthood also did not impact instrumental response acquisition. (e) Unlike with adolescent CORT exposure, however, both groups inhibited a response that was unlikely to be reinforced in a goal-directed fashion. *n* = 7/group. (f) Additionally, all mice inhibited responding following prefeeding with the reinforcer pellets (“devalued”), relative to prefeeding with chow (“non-devalued”), regardless of age of CORT exposure. (g) Another group of adolescent CORT-exposed mice was allowed a longer (4-wk) washout period in order to match the timing of testing in adult CORT-exposed mice. Mice acquired the responses without group differences. (h) Response preferences were intact in test 1, as above. During test 2, mice were initially able to differentiate between the responses that were likely versus unlikely to be reinforced, but response preference decayed in the CORT-exposed mice. *n* = 11–12/group. Bars/symbols = means + SEMs, * *p* < 0.05, ** *p* < 0.001 versus nondegraded or main effects, as indicated. Raw data for this figure can be found in S1 Data. CORT, corticosterone; FR1, fixed ratio 1; RI, random interval.

Having characterized our model, we next determined whether the same subchronic CORT exposure procedure would impact habit biases. We first trained control and CORT-exposed mice to nose poke 2 separate recesses for food reinforcers. We detected no side biases nor group differences in instrumental response acquisition rates (*F*_(1,15)_ = 2.7, *p* = 0.12; interaction *F*_(9,135)_ = 1.6, *p* = 0.12) (Fig 2a).

We next decreased the likelihood that 1 response would be reinforced. A “goal-directed” response strategy is to then preferentially engage the remaining behavior, which remains likely to be reinforced, while habit-based responding is insensitive to action–outcome contingency [23]. In this case, mice engage both responses (both “non-degraded” and “degraded”) equivalently. Following an initial test, both groups inhibited the response that was unlikely to be reinforced in a goal-directed (nonhabitual) fashion (main effect *F*_(1,15)_ = 11.6, *p* = 0.004; no interaction) (test 1, Fig 2b). Response rates were also lower overall in the CORT-exposed mice, consistent with diminished break point ratios in the progressive ratio test (Fig 1c).

With additional training using random interval (RI) schedules of reinforcement that can bias responding towards habits, mice with a history of CORT exposure indeed assumed habit-based strategies, failing to differentiate between the behaviors that were more (or less) likely to be reinforced. Meanwhile, control mice differentiated between the responses, retaining goal-oriented response strategies (interaction *F*_(1,13)_ = 6.0, *p* = 0.03) (test 2, Fig 2b). Thus, subchronic CORT exposure in adolescence causes a bias towards habit formation in adulthood. Notably, we discovered the same patterns when we tested female, rather than male, mice (S2 Fig).

In separate mice, test 2 occurred in a distinct context. In this case, both groups generated the response that was likely to be reinforced (main effect *F*_(1,12)_ = 16.7, *p* = 0.001; no interaction) (Fig 2c). Thus, adolescent CORT-induced habits were context dependent.

Next, we assessed whether subchronic CORT exposure similarly impacted adult mice. CORT exposure decreased body weights as expected (Table 2). While instrumental response rates during training were generally lower than in our younger cohorts, control versus CORT-exposed groups did not differ (*F*s < 1) (Fig 2d). And unlike with subchronic CORT exposure in adolescence, both groups consistently generated the response most likely to be reinforced in a goal-directed fashion (main effect test 1: *F*_(1,12)_ = 8.4, *p* = 0.01; main effect test 2: *F*_(1,12)_ = 36, *p* < 0.001; no interactions) (Fig 2e). Thus, adolescents were more vulnerable to the habit-inducing influence of elevated glucocorticoids.

**Table 2:** Body weights following CORT, stress, and 7,8-DHF. (**Row A**). The change in body weight across days of mice exposed to CORT or water during early adolescence depended on CORT status (day × CORT interaction *F*_(5,75)_ = 4.1, *p* = 0.003), but post hoc tests were nonsignificant. **(Row B)** In adult mice, body weight change across days also depended on CORT status (day × CORT interaction *F*_(5,60)_ = 12.1, *p* < 0.001), but post hoc tests were nonsignificant. **(Row C)** Body weight of mice exposed to repeated forced swimming or control manipulation during early adolescence increased across days (main effect of day *F*_(5,65)_ = 22.7, *p* < 0.001), with no group differences (main effect and interaction *p* > 0.05). **(Row D)** For mice exposed to CORT or water during adolescence and treated with 7,8-DHF (0.0, 3.0, or 10.0 mg/kg), the change in body weight across days depended on CORT status (day × CORT interaction *F*_(8,240)_ = 18.1, *p* < 0.001], with CORT impairing normal weight gain (days on which control and CORT groups significantly differed are indicated by asterisks, as determined by post hoc tests). There were no effects of 7,8-DHF treatment alone, nor any interactions (*p* > 0.05). **(Row E)** The average body weight of female mice exposed to CORT or water during early adolescence and treated with 0.0 or 3.0 mg/kg of 7,8-DHF increased across days (main effect *F*_(8,168)_ = 42.1, *p* < 0.001), with no group differences (*p* > 0.05). Shaded cells indicate the period of 7,8-DHF or vehicle administration. Unit of measure is grams. ** refers to interaction effects; * refers to post hoc comparisons (both *p* < 0.05). 7,8-DHF dosing (in mg/kg) is indicated in parentheses. Raw data for this table can be found in S1 Data.

			Day 1	Day 3	Day 5	Day 7	Day 9	Day 11	Day 13	Day 15	Day 17
			Mean±SEM	Mean±SEM	Mean±SEM	Mean±SEM	Mean±SEM	Mean±SEM	Mean±SEM	Mean±SEM	Mean±SEM
***A***. *Adolescence*		control	12.6 ± 0.96	14.0 ± 0.99	14.8 ± 0.93	15.8 ± 0.81	16.9 ± 0.63	17.7 ± 0.44	n/a		
		CORT**	13.6 ± 0.33	15.4 ± 0.33	15.0 ± 0.27	16.1 ± 0.27	16.6 ± 0.20	16.9 ± 0.18	n/a		
***B***. *Adulthood*		control	22.8 ± 1.01	23.3 ± 1.10	23.6 ± 1.10	23.9 ± 1.07	24.1 ± 1.07	24.1 ± 1.13	n/a		
		CORT **	21.2 ± 0.95	21.3 ± 0.92	21.3 ± 0.98	21.3 ± 0.92	21.3 ± 0.94	21.2 ± 0.93	n/a		
***C***. *Adolescence*		control	15.1 ± 0.59	15.7 ± 0.49	16.6 ± 0.24	16.8 ± 0.34	17.1 ± 0.39	16.6±0.35	n/a		
		stress	15.8 ± 0.60	16.1 ± 0.47	16.6 ± 0.53	16.6 ± 0.43	16.8 ± 0.45	16.8±0.38	n/a		
***D***. *Adolescence* (*7*,*8-DHF*)–*M*	control	veh	16.5 ± 0.48	17.5 ± 0.29	17.8 ± 0.29	18.3 ± 0.34	18.6 ± 0.31	18.9 ± 0.32	18.8 ± 0.41	18.9 ± 0.37	19.6 ± 0.30
		DHF(3)	16.5 ± 1.08	16.7 ± 0.83	17.2 ± 0.56	17.8 ± 0.52	18.3 ± 0.43	18.7 ± 0.39	18.6 ± 0.46	19.2 ± 0.40	19.4 ± 0.42
		DHF(10)	16.5 ± 0.90	17.5 ± 0.76	17.8 ± 0.62	18.1 ± 0.54	18.4 ± 0.55	18.7 ± 0.56	18.8 ± 0.60	19.4 ± 0.59	19.6 ± 0.52
	CORT **	veh	15.9 ± 0.47	16.5 ± 0.35	*16.1 ± 0.34	*16.1 ± 0.37	*16.1 ± 0.36	*16.3 ± 0.34	*16.1 ± 0.32	*16.9 ± 0.33	*17.7 ± 0.34
		DHF(3)	16.6 ± 0.72	16.7 ± 0.69	*16.8 ± 0.62	*16.7 ± 0.49	*16.5 ± 0.52	*16.8 ± 0.50	*16.8 ± 0.51	*17.4 ± 0.59	*18.4 ± 0.55
		DHF(10)	16.9 ± 0.79	17.3 ± 0.38	*17.2 ± 0.37	*17.3 ± 0.39	*17.3 ± 0.48	*17.4 ± 0.47	*17.3 ± 0.49	*18.0 ± 0.51	*19.0 ± 0.53
***E***. *Adol*. (*DHF*)–*F*	control	veh	13.5 ± 0.31	13.4 ± 0.26	13.6 ± 0.17	14.4 ± 0.15	14.2 ± 0.14	14.4 ± 0.16	14.6 ± 0.18	14.6 ± 0.18	14.8 ± 0.15
	CORT	veh	13.5 ± 0.36	13.4 ± 0.28	13.5 ± 0.22	14.1 ± 0.23	14.3 ± 0.26	14.6 ± 0.24	14.4 ± 0.38	14.9 ± 0.40	15.0 ± 0.37
		DHF(3)	13.2 ± 0.42	13.5 ± 0.32	13.8 ± 0.24	14.3 ± 0.27	14.4 ± 0.31	14.7 ± 0.26	14.8 ± 0.31	15.4 ± 0.32	15.4 ± 0.33

**Abbreviations**: Adol., adolescence; 7,8-DHF and DHF, 7,8-dihydroxyflavone; CORT, corticosterone; F, females; M, males; veh, vehicle

Insensitivity to instrumental contingencies is commonly associated with insensitivity to reinforcer value. However, when we tested the same mice for sensitivity to reinforcer devaluation, we found no impairment in response inhibition following ad libitum access to the reinforcer pellets prior to test (“devalued” condition), relative to prefeeding with regular chow (“non-devalued” condition) (main effect *F*_(1,26)_ = 28, *p* < 0.001; no interaction) (Fig 2f). Thus, subchronic CORT exposure during adolescence, but not adulthood, induced failures in selecting actions based on their consequences. This failure was context dependent, while value-based action selection was intact.

In a final experiment, we exposed adolescent mice to CORT, then trained them to respond for food reinforcers following a prolonged, 4-wk washout period. This procedure doubled the “recovery” period following CORT and matched the age of testing in our adult CORT-exposed population. Mice acquired the nose poke responses without group differences (*F*_(1,21)_ = 2.7, *p* = 0.12; interaction *F*s < 1.2) (Fig 2g). Mice were sensitive to action–outcome contingency degradation at test 1, as in all other groups (main effect *F*_(1,21)_ = 5.8, *p* = 0.03; no interaction) (Fig 2h). In a second test, CORT-exposed mice again preferentially generated the response most likely to be reinforced; however, this preference decayed (group × response × time interaction *F*_(1,20)_ = 4.2, *p* = 0.05) (Fig 2h). Thus, adolescent CORT-exposed mice recovered some function with a prolonged washout period (as opposed to our shorter washout period tested above), but habitual response biases remained detectable nearly 1 mo following exposure.

### CORT shifts corticolimbic trkB/trkB.t1 ratios and reduces p-ERK42/44 in the vHC

**Fig 3:**
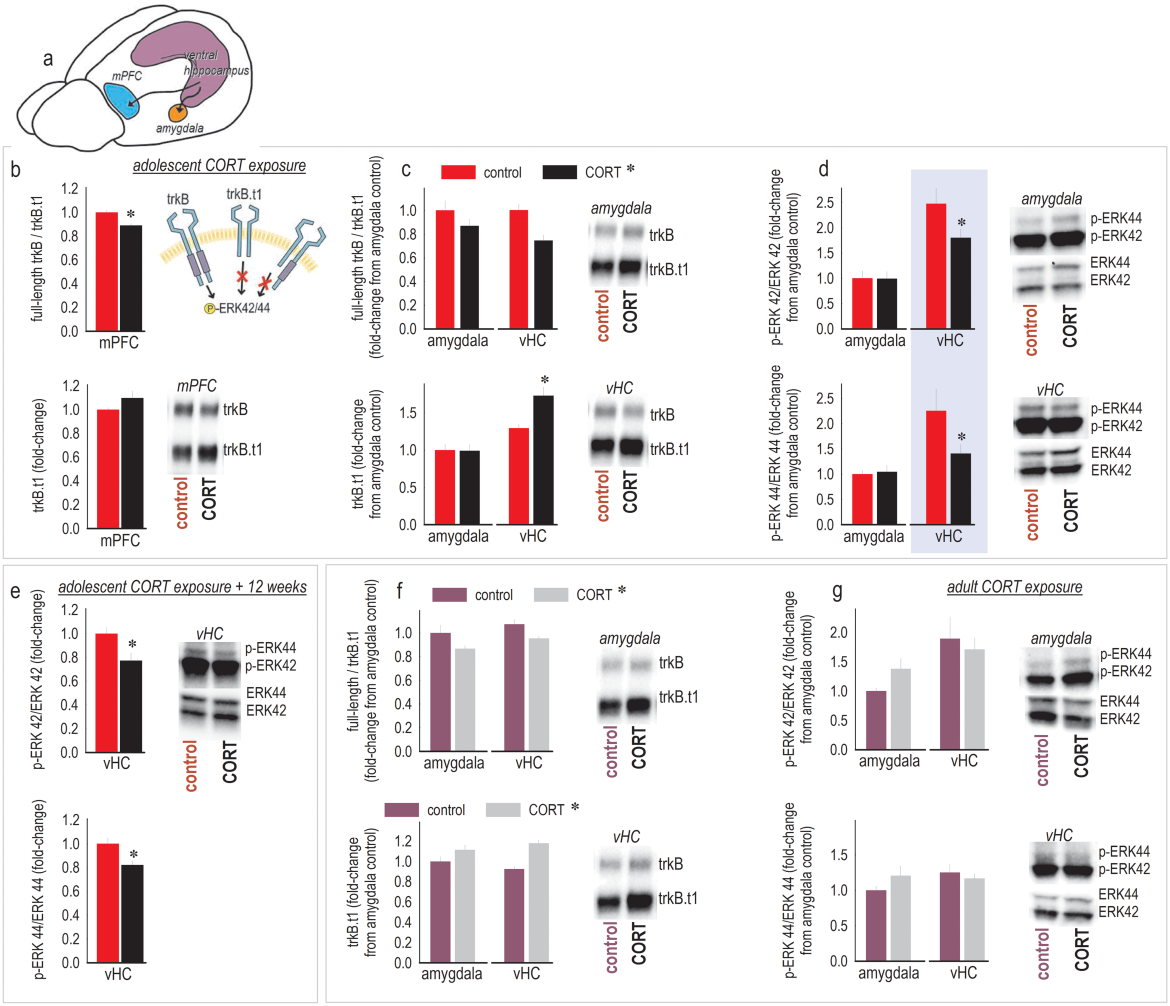
Adolescent CORT exposure regulates cortico-limbic trkB and ERK42/44 phosphorylation. (a) Based on our findings, we profiled the neurobiological effects of CORT in a mPFC–vHC–amygdala circuit. (b) Brains were first collected at the end of the CORT exposure period. **Top**: Adolescent CORT exposure decreased the ratio of full-length to truncated trkB in the mPFC, which would decrease the ability of full-length trkB to initiate intracellular signaling events, illustrated at right. **Bottom**: trkB.t1 levels alone did not significantly differ. (c) **Top**: Adolescent CORT exposure also decreased the ratio of full-length to truncated trkB in the vHC and amygdala. **Bottom**: In the vHC, this was accompanied by an increase in overall trkB.t1 levels. (d) **Top**: p-ERK42 levels were higher overall in the vHC than in the amygdala, and CORT reduced vHC p-ERK42 (in planned comparisons, see also Table 3). **Bottom**: The same pattern was detected for p-ERK44. (e) To determine whether this effect was long lasting, we assessed ERK42/44 phosphorylation 12 wk following adolescent CORT exposure. **Top**: p-ERK42 was reduced in the vHC. **Bottom**: p-ERK44 was also blunted. (f) Next, we tested the effects of subchronic CORT in adult mice. **Top**: CORT decreased trkB:trkB.t1 in the amygdala and vHC, as in adolescent mice. **Bottom**: trkB.t1 was also elevated. (g) Despite these modifications, ERK42/44 phosphorylation was not impacted. Representative blots are adjacent throughout. These and additional analyses are summarized in Table 3. *n* = 4–10/group throughout. Bars/symbols = means+SEMs, * *p* < 0.05 versus control within the same brain region. When in the legend, asterisks indicate main effects of CORT. Raw data for this figure can be found in S1 Data. CORT, corticosterone; ERK42/44, extracellular signal-regulated kinase 42/44; mPFC, medial prefrontal cortex; p-ERK, phosphorylated ERK; trkB, tyrosine kinase receptor B; trkB.t1, truncated trkB; vHC, ventral hippocampus.

The transition from goal-directed to habit-based modes of response has been characterized as a decline in behavioral control by specific prefrontal cortex (PFC)-limbic structures (e.g., Fig 3a), in favor of sensorimotor circuits [11–13,23–25]. Within the hippocampus, the ventral compartment provides the primary inputs to the PFC [26]. For these reasons, we assessed levels of the stress-sensitive neurotrophin receptor trkB and phosphorylation of its substrate ERK42/44 in a mPFC–vHC–amygdala–striatal network. Results are reported in Table 3. Key findings are also displayed graphically. Specifically, adolescent CORT exposure decreased the ratio of full-length trkB/trkB.t1 in the mPFC (*t*_12_ = 4.3, *p* < 0.001) (Fig 3b). This pattern was not anatomically selective—in both the vHC and amygdala, developmental CORT also decreased trkB/trkB.t1 ratios (main effect of CORT *F*_(1,23)_ = 10.7, *p* = 0.003) (Fig 3c, top), as was also observed in the ventral striatum (*t*_10_ = 3.9, *p* = 0.003) (Table 3). CORT elevated overall levels of trkB.t1 in the vHC (interaction *F*_(1,21)_ = 5.8, *p* = 0.025) (Fig 3c, bottom) and ventral striatum (*t*_10_ = −2.3, *p* = 0.046) (Table 3), but only in the vHC were levels of phosphorylated (active) ERK42/44 also decreased by CORT (Fig 3d; Table 3). Of note, phosphorylated ERK (p-ERK) levels were also generally higher in the vHC than the amygdala (p-ERK42 *F*_(1,18)_ = 16.4, *p* < 0.001; p-ERK44 *F*_(1,18)_ = 8.5, *p* = 0.009) (Fig 3d).

**Table 3:** Protein levels following subchronic (11 d) CORT exposure during early adolescence. Levels of full-length and truncated trkB receptor isoforms and phosphorylated ERK42/44 were quantified in various brain regions at the end of the CORT exposure period in adolescents. Values represent the % change relative to control animals.

		vHC	Amygdala	mPFC	DMS	VS
*CORT as % of control*	trkB/trkB.t1	74.4*	86.9*	88.8*	99.3	84.7*
	trkB.t1	133.9*	99.1	109.7	101.5	119.4*
	trkB	92	90.4	97	102	100.9
	p-ERK42	72.8*	99.6	113.6	99.6	136.9
	p-ERK44	62.5*	104.7	104.6	99.3	115.5

* *p* < 0.05 in 2-tailed *t* tests. Abbreviations: vHC, ventral hippocampus; mPFC, medial prefrontal cortex; DMS, dorsomedial striatum; VS, ventral striatum

To determine whether this effect was long lasting, we immunoblotted for p-ERK42/44 in the vHC 12 wk following adolescent CORT exposure, again revealing lower phosphorylated levels of both ERK isoforms (p-ERK42: *t*_18_ = 2.7, *p* = 0.01; p-ERK44: *t*_18_ = 3, *p* = 0.008) (Fig 3e).

We next tested adult mice exposed to CORT. CORT again decreased amygdalo–hippocampal ratios of full-length trkB/trkB.t1 (main effect *F*_(1,18)_ = 9.4, *p* = 0.007) and also elevated trkB.t1 (main effect *F*_(1,20)_ = 21.2, *p* < 0.001) (Fig 3f); however, we identified no effect of CORT on p-ERK42/44 (main effects and interactions *p* > 0.18) (Fig 3g). Thus, a unique consequence of subchronic CORT exposure in adolescence appears to be the elevation of trkB.t1 and concurrent suppression of p-ERK42/44 within the vHC.

### Blockade of CORT-induced habits and amotivation by trkB stimulation

**Fig 4:**
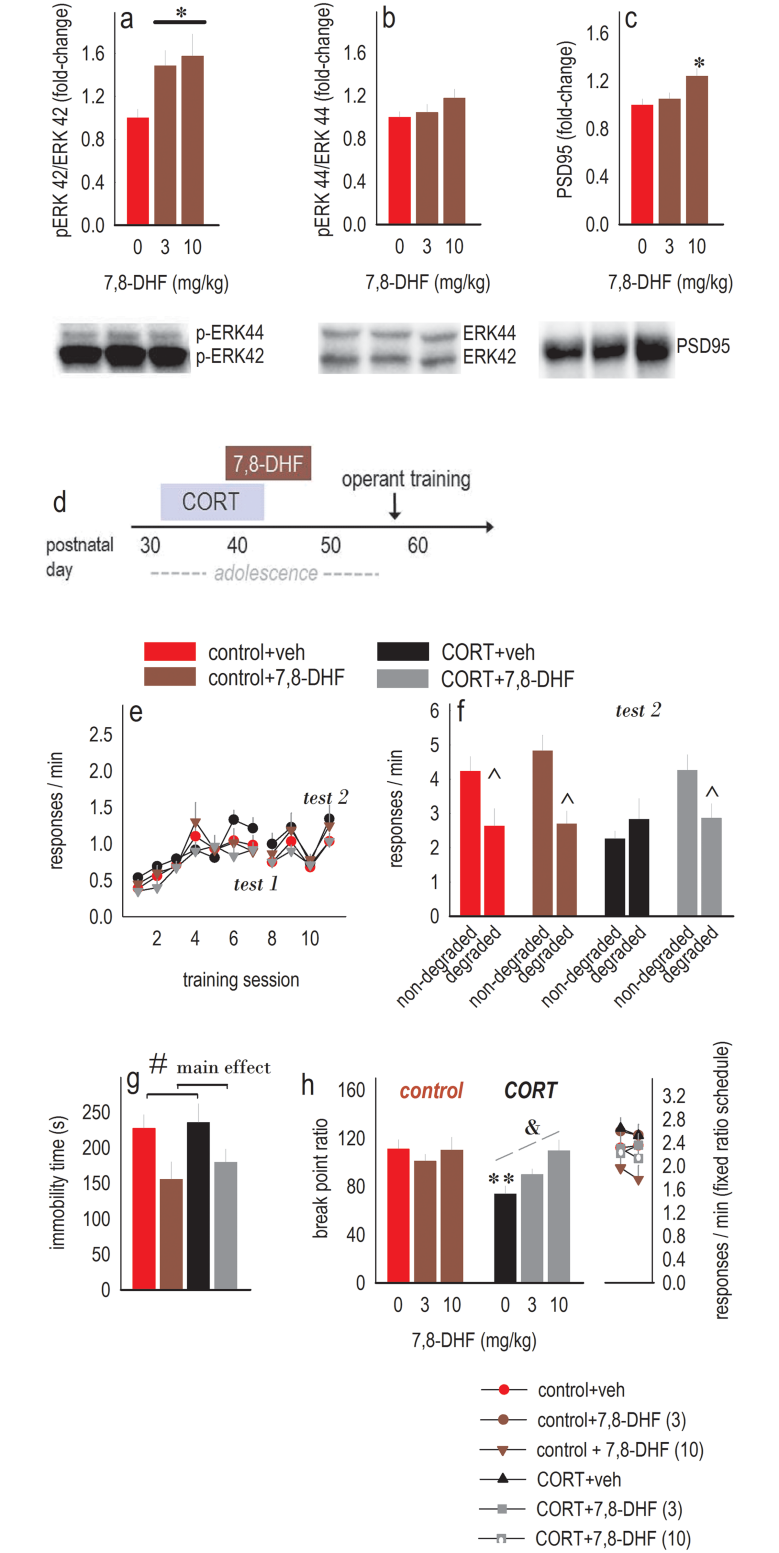
Durable blockade of CORT-induced habits and depression-like behavior. (a) Repeated 7,8-DHF treatment increased p-ERK42 in the vHC (b) but had no effects on p-ERK44. (c) At the highest dose tested, levels of the synaptic marker PSD95 were also increased relative to the control and 3 mg/kg groups. Representative blots are below. *n* = 9–14/group. (d) Experimental timeline. (e) Mice developed food-reinforced instrumental responses without differences between groups. Response acquisition curves represent both responses/min, and breaks in the acquisition curves represent tests for sensitivity to instrumental contingency degradation. (f) As shown in the prior figures, CORT-exposed mice developed habit-based response strategies. 7,8-DHF at 3 mg/kg blocked these habits, as indicated by preferential engagement of the response most likely to be reinforced in the CORT+7,8-DHF group. *n* = 5–6/group. (g) 7,8-DHF–treated mice were also less immobile in the forced swim test, a durable antidepressant-like effect. *n* = 7/group. (h) Furthermore, 7,8-DHF dose-dependently blocked CORT-induced deficiencies in break point ratios (while having no effects on responding when a fixed ratio 1 schedule was applied; symbols at right). *n* = 6–13/group. Bars/symbols = means + SEMs, * *p* < 0.05, ** *p* <0.001 following CORTx7,8-DHF interaction, ^ *p* < 0.001 following response choice interactions, & *p* = 0.002 versus CORT alone, # *p* < 0.05 main effect of 7,8-DHF (no interaction). Raw data for this figure can be found in S1 Data. 7,8-DHF, 7,8-dihydroxyflavone; CORT, corticosterone; ERK42/44, Extracellular signal-Regulated Kinase 42/44; p-ERK, phosphorylated ERK; PSD95, postsynaptic density 95; vHC, ventral hippocampus.

Next, we attempted to block adolescent CORT-induced habits with the trkB agonist 7,8-dihydroxyflavone (7,8-DHF). We first aimed to identify a dose range that stimulated p-ERK42/44 in the vHC. Three and 10 mg/kg, intraperitoneal (i.p.), induced ERK42 phosphorylation (*F*_(2,38)_ = 3.9, *p* = 0.03) (Fig 4a), though p-ERK44 levels were not affected (*F*_(2,38)_ = 1.7, *p* = 0.2) (Fig 4b). 7,8-DHF also blocked the chronic p-ERK42 deficit due to CORT exposure (Table 4) while having no long-term consequences for adrenal and thymus gland weights (S1 Table), and the 10 mg/kg dose increased levels of the postsynaptic marker, postsynaptic density 95 (PSD95) (*F*_(2,24)_ = 4.8, *p* = 0.02) (Fig 4c).

**Table 4:** Protein levels in the vHC of adult mice exposed to CORT during adolescence ± 7,8-DHF (10 mg/kg). 7,8-DHF influenced p-ERK42/ERK42 in CORT-exposed mice (CORT × 7,8-DHF interaction *F*_(1,28)_ = 4.2, *p* = 0.05). Post hoc tests indicated that control + veh group significantly differed from CORT + veh group as reported in independent experiments (see Table 3 and Fig 3) (indicated by asterisks, * *p* < 0.05), but 7,8-DHF eliminated this difference. CORT decreased p-ERK44/ERK44, but there was no effect of 7,8-DHF (main effect CORT *F*_(1,27)_ = 8.1, *p* = 0.008, no interaction). Values indicate fold change from control + veh group. Raw data for this table can be found in S1 Data.

			p-ERK42/ERK42 Mean ± SEM	p-ERK44/ERK44 Mean ± SEM
*Adolescence* (*7*,*8-DHF*)	control	veh	*1.00 ± 0.05	1.00 ± 0.05
		DHF(10)	0.90 ± 0.12	1.05 ± 0.07
	CORT	veh	*0.77 ± 0.06	0.82 ± 0.03
		DHF(10)	1.11 ± 0.21	0.93 ± 0.04

7,8-DHF and DHF, 7,8-dihydroxyflavone; CORT, corticosterone; ERK42/44, Extracellular signal-Regulated Kinase 42/44; p-ERK, phosphorylated ERK

Next, separate mice were exposed to CORT during early adolescence, and half were treated with 7,8-DHF (3 mg/kg) from P39–47, overlapping with the end of the CORT exposure period and extending into late adolescence [20] (Fig 4d). As adults, mice acquired the nose poke responses with no group differences (main effect CORT and 7,8-DHF *F*s < 1; CORT × 7,8-DHF interaction *F*_(1,19)_ = 3.8, *p* = 0.065; all other interactions *F*s ≤ 1.1) (Fig 4e). Following an initial instrumental contingency degradation test, all groups preferentially generated the response most likely to be reinforced in a goal-directed fashion as in our experiments described above (main effect *F*_(1,20)_ = 24.6, *p* < 0.001; no interaction) (test 1, not shown). With more training, CORT-exposed mice developed habit-based behavior, also as expected, but critically, 7,8-DHF blocked CORT-induced habit biases (CORT × response interaction *F*_(1,19)_ = 7.2, *p* = 0.015; 7,8-DHF × response interaction *F*_(1,19)_ = 5.3, *p* = 0.03) (test 2, Fig 4f). This effect of 7,8-DHF was also detectable in female mice (S2 Fig).

We next tested these mice in the forced swim test. As in Fig 1, prior CORT did not impact immobility in the absence of stressor exposure in adulthood. However, a history of 7,8-DHF treatment reduced time spent immobile, an antidepressant-like effect that was notably detectable multiple weeks following treatment (main effect of 7,8-DHF *F*_(1,20)_ = 8.3, *p* = 0.008; no interaction) (Fig 4g). The reduction in time spent immobile could not obviously be attributable to general hyperactivity following 7,8-DHF treatment (S2 Table).

We also quantified responding on a progressive ratio schedule of reinforcement. 7,8-DHF dose-dependently blocked CORT-induced deficits in break point ratios, and this blockade was detectable when either total responses or break points were compared (interactions *F*_(2,56)_ = 3.9, *p* = 0.03; *F*_(2,54)_ = 3.4, *p* = 0.04). Break points are shown (Fig 4h).

### Recapitulating the long-term effects of adolescent CORT exposure with *Trkb*.*t1* overexpression

**Fig 5:**
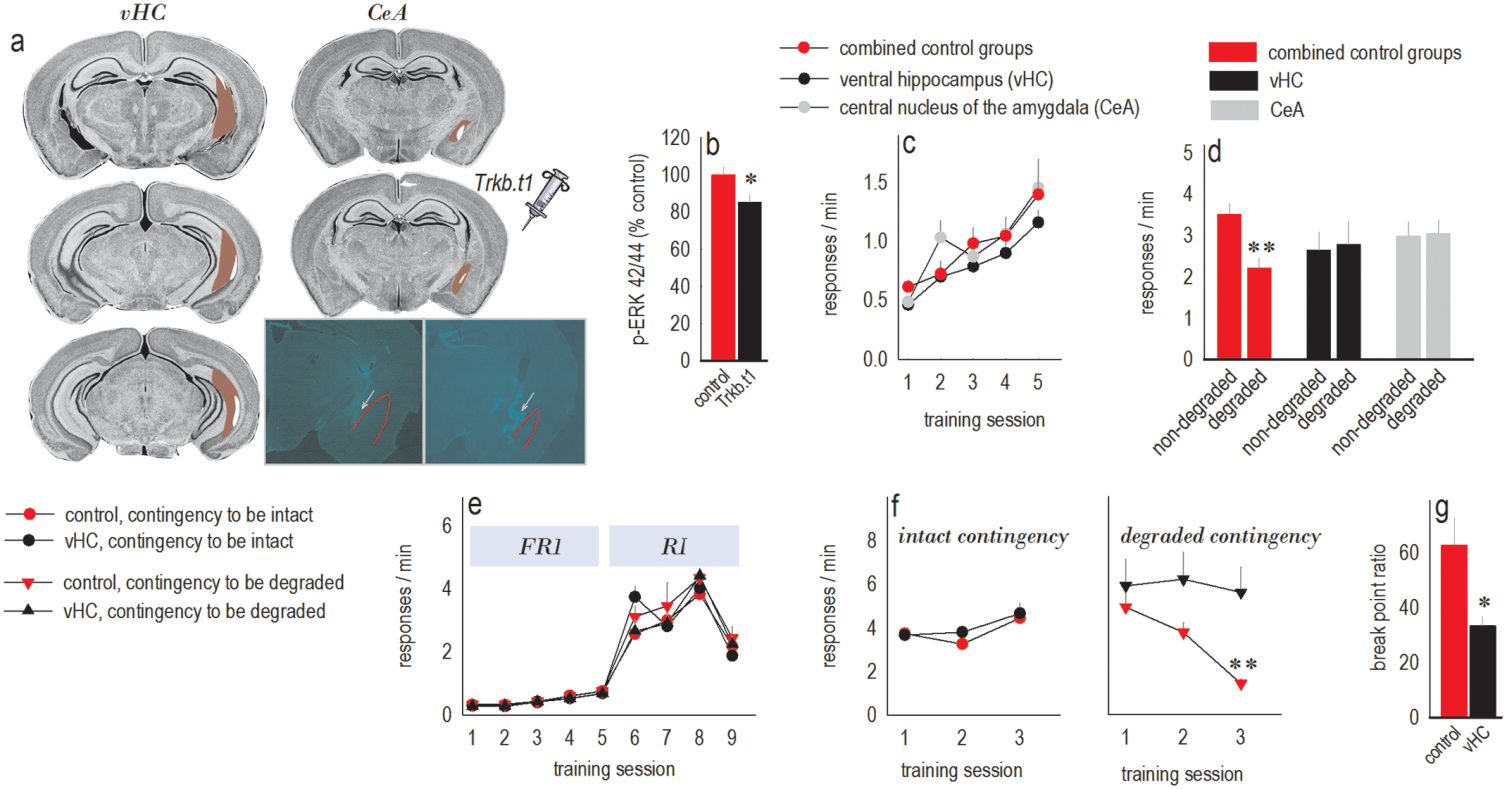
Overexpression of truncated trkB induces habits, makes them harder to “break,” and causes depression-like behavior. (a) We infused a lentivirus expressing truncated trkB (*Trkb*.*t1*) or GFP into the vHC and CeA. Large (red) and small (white) infusion sites are represented on images from the Mouse Brain Library [27]. All infusion sites are described in S3 Fig. At bottom right, representative lentiviral GFP expression in the CeA is shown (at gray arrows). The external capsule is highlighted in red, and the image is intentionally overexposed. (b) In *Trkb*.*t1*-expressing mice, p-ERK42/44 immunoreactivity was diminished at the infusion site. *n* = 7–8/group. (c) All mice acquired the instrumental responses. Response acquisition curves represent both responses/min. (d) *Trkb*.*t1* in the vHC and CeA blocked sensitivity to instrumental contingency degradation. Thus, selective *Trkb*.*t1* overexpression recapitulated the long-term effects of adolescent CORT exposure. *n* = 6–8/*Trkb*.*t1* group; total control = 23. (e) Another group of mice was first trained using a fixed ratio schedule of reinforcement. Then, to build on our findings reported in (d), an RI schedule of reinforcement was applied, with no interruption in training and no differences in responding between groups. (f) In reaction to repeated instrumental contingency degradation training, control mice inhibited a response that was unlikely to be reinforced, their habits “breaking” (“degraded contingency,” right). By contrast, mice with vHC *Trkb*.*t1* failed to inhibit responding. Response rates associated with an intact contingency were unaffected (left). Response rates are represented on 2 plots in the interest of clarity but were compared together by ANOVA. *n* = 9–10/group. (g) Finally, vHC *Trkb*.*t1* overexpression also decreased responding on a progressive ratio schedule of reinforcement in adulthood, again recapitulating the long-term effects of adolescent CORT exposure. *n* = 9/group. Bars/symbols = means + SEMs, * *p* < 0.05, ** *p* ≤ 0.004. Raw data for this figure can be found in S1 Data. CeA, central nucleus of the amygdala; ERK42/44, Extracellular signal-Regulated Kinase 42/44; p-ERK, phosphorylated ERK; trkB, tyrosine kinase receptor B; trkB.t1, truncated trkB; vHC, ventral hippocampus.

To summarize, adolescent CORT exposure increases levels of trkB.t1 and decreases p-ERK42/44 in the vHC and also induces biases towards habit-based behaviors. These biases are blocked by the putative trkB agonist 7,8-DHF at doses that increase p-ERK42 in vHC. To determine whether selectively elevating trkB.t1 and decreasing p-ERK42/44 in the vHC is sufficient to recapitulate the behavioral effects of CORT exposure, we overexpressed *Trkb*.*t1* in the vHC and a major projection target, the central nucleus of the amygdala (CeA) (Fig 5a). vHC-targeted infusions were mostly restricted to the ventral Cornu amonis (CA) 1 region, with some spread into the intermediate hippocampus (Fig 5a; see also S3 Fig). Amygdala-targeted infusions were largely contained within the CeA as intended (Fig 5a; see also S3 Fig). Seven mice were excluded due to mistargeted infusions infecting white matter tracts, and control green fluorescent protein (GFP)-expressing mice did not differ and were combined.

At the infusion site, p-ERK42/44 levels were reduced by 15% in *Trkb*.*t1*-expressing mice relative to mice expressing GFP (*t*_13_ = 2.6, *p* = 0.02) (Fig 5b), mimicking the long-term consequences of adolescent CORT exposure (compare to Fig 3d). Mice acquired the food-reinforced instrumental responses without group differences (*F*s ≤ 1) (Fig 5c). GFP-expressing control mice were sensitive to instrumental contingency degradation, preferentially engaging the response most likely to be reinforced. By contrast, *Trkb*.*t1* overexpression induced inflexible habits, indicated by a failure to respond in a selective fashion following instrumental contingency degradation (interaction *F*_(2,34)_ = 5.6, *p* = 0.008) (Fig 5d). Thus, *Trkb*.*t1* overexpression recapitulated the long-term effects of adolescent CORT exposure.

Next, we expanded these studies, deviating from our protocol used thus far, to assess whether *Trkb*.*t1* overexpression in the vHC also interfered with the ability to “break” habits. We accordingly trained separate mice using an RI schedule of reinforcement. Instrumental response acquisition curves are segregated according to whether the action–outcome contingency associated with each response would ultimately remain intact or be “degraded,” highlighting equivalent response rates throughout (*F*s < 1) (Fig 5e).

We modified our typical instrumental contingency degradation procedure to determine whether responses could be inhibited once habits formed. Specifically, we exposed mice to alternating training sessions in which 1 response was reinforced or the contingency between the other response and its outcome was degraded. Initially, mice responded equivalently during these 2 types of training sessions, exhibiting habit-based behavior. With repeated testing, control mice were ultimately able to inhibit the response that was unlikely to be reinforced. By contrast, response strategies in *Trkb*.*t1*-overexpressing mice were unchanged and habit-based (group by contingency interaction *F*_(1,32)_ = 6.6, *p* = 0.02) (Fig 5f). Thus, *Trkb*.*t1* overexpression caused significant behavioral inflexibility.

Finally, we also confirmed that vHC *Trkb*.*t1* overexpression decreased break point ratios in a progressive ratio test (*t*_16_ = 2.8, *p* = 0.01) (Fig 5g), as with adolescent CORT exposure. This finding is consistent with evidence that vHC-targeted knockdown of the trkB ligand BDNF also induces depression-like behavior [28].

## Discussion

Early-life stress is associated with inflexible habit-based behavior in humans [1,5], and in both humans and rodents, glucocorticoid exposure induces habit biases [3,4]. Despite these homologies, mechanisms are largely uncharacterized. We report that CORT exposure in mice during a period equivalent to early adolescence in humans induces a bias towards habit-based behaviors; a shift in the balance between full-length, active trkB and an inactive, truncated form of the receptor throughout multiple corticolimbic brain regions; and p-ERK42/44 deficits selectively in the vHC (summarized in Table 3). Viral-mediated overexpression of *Trkb*.*t1* selectively in the vHC decreases local p-ERK42/44 and causes habit-based behavior. Meanwhile, stimulating trkB-ERK42/44 during adolescence blocks CORT-induced habits and has antidepressant-like effects that are detectable well after the treatment period, revealing an early-life sensitive period when trkB–ERK42/44 tone has long-term behavioral consequences.

We first validated our CORT exposure procedure, revealing elevated blood serum levels during the active dark cycle that were comparable to CORT levels following forced swim stress. Meanwhile, blood serum CORT during the inactive light cycle (when mice are sleeping and not consuming CORT) was unaffected. Adolescent CORT exposure additionally reduced progressive ratio break points, as also occurs in cases of adult CORT exposure [3,29–31]. This behavioral phenotype has been likened to amotivation in depression and is consistent with considerable evidence that a history of stressor exposure is a primary predictor of depression [32].

Unlike chronic CORT (e.g., [29]), subchronic CORT (here) did not induce immobility in the forced swim test, but reactivity to an acute stressor was impacted. Specifically, water deprivation induced mobility in control mice (see also [33]), a so-called “active coping” response [22]. Meanwhile, CORT-exposed mice maintained high levels of immobility, a “passive coping” response that has also been interpreted as depression-like [34]. Dendritic spines were also eliminated in the mPFC, a common reaction to stressor and CORT exposure in mature rodents [21,35]. These outcomes may be related, given that switching between “active” and “passive” swimming phenotypes is dependent upon stimulation of mPFC projections to brainstem targets [36].

### Adolescent CORT exposure has persistent neurobehavioral consequences

Adolescent CORT exposure also induced a long-term bias towards habit-based response strategies. Specifically, mice were initially able to select actions (left/right nose poke) based on their consequences (food), but with repetition, these behaviors assumed habitual qualities such that they were insensitive to response–outcome contingency. Subchronic CORT exposure did not impact adult mice, indicating that adolescents are more vulnerable to developing CORT-induced habits. Moreover, when we doubled the “recovery” period duration following adolescent CORT exposure, all mice could initially select actions based on their consequences, but response preferences faded over time in CORT-exposed mice. We interpret this as uncertainty in response selection, resulting in a deferral to familiar, habit-based behaviors that are insensitive to response–outcome associations.

Behavioral insensitivity to response–outcome contingency is often associated with insensitivity to reinforcer value [23]. This was not the case here, however, in that all mice reduced response rates following prefeeding with the reinforcer pellets, which decreases their value. Why might this be? One possibility is that subchronic CORT exposure particularly impacted hippocampal function. Lesions of the entorhinal cortex, a primary input to the hippocampus, reduce sensitivity to response–outcome contingencies but not reinforcer value, as with CORT here [37,38]. This may be because organisms form an association between the context and response–outcome contingency during training. When that contingency is later violated, the hippocampus detects the discrepancy between “the context where I typically work for reward” and noncontingent pellet delivery and facilitates response inhibition. This model predicts that response strategies should be intact if instrumental contingency degradation occurs in a contextually distinct environment relative to the training environment, which was indeed the case here. In contrast, behavioral sensitivity to reinforcer devaluation is context-independent because it relies on an animal’s ability to prospectively calculate reinforcer value. Accordingly, it is unaffected by entorhinal cortex lesions [37,38], while lesions/inactivation of other structures, such as the dorsal mPFC, basolateral amygdala, and dorsomedial striatum impair sensitivity to both response–outcome contingency and reinforcer value [23].

Based on these findings, we next quantified levels of the stress-sensitive neurotrophin receptor trkB in the vHC and other regions that counter habit-based behavior (dorsal mPFC, amygdala, dorsomedial striatum, and ventral striatum; see [11–13,23]). CORT caused widespread modifications in the ratio of full-length:truncated isoforms, favoring the inactive isoform. (Indeed, of the brain regions tested, only the dorsomedial striatum was spared.) This is significant because trkB.t1 dimerization with full-length trkB reduces the receptor’s ability to stimulate ERK42/44, PI3-kinase, and PLCγ. trkB.t1 is also linked to neurodegeneration [39] and excitotoxicity [40]. Additionally, increasing trkB.t1 decreases cell-surface levels of full-length trkB, further reducing opportunities for trkB-mediated signaling [41].

TrkB.t1 levels remained particularly robust in the vHC even following the CORT exposure period. These findings are in general agreement with prior investigations using social [42] (though not physical [43]) stress. Also, transgenic mice overexpressing *Trkb*.*t1* are insensitive to classical antidepressants [44], further motivating us to investigate whether direct trkB stimulation could have antidepressant-like properties following CORT. Indeed, 7,8-DHF given during adolescence (P39–47) corrected CORT-induced habit behavior and amotivation and increased mobility in the forced swim test, an antidepressant-like effect. 7,8-DHF derivatives also have antidepressant-like consequences in adult mice [45,46] and restore the reinforcing properties of cocaine following stress [47], but ours is the first evidence, to our knowledge, of antidepressant-like efficacy in adolescents. And importantly, effects were detectable well beyond the treatment period.

How might adolescent 7,8-DHF treatment confer long-term benefits? Enhancing trkB-mediated signaling could correct the suppressive effects of stressor or CORT exposure on neurogenesis in the vHC or restore BDNF–trkB interactions in the hippocampus, both of which would be associated with antidepressant-like efficacy [29,48,49]. Also of note, vHC innervation of the anterior mPFC develops during adolescence [50]. We found that adolescent CORT exposure caused dendritic spine elimination in the anterior mPFC. Additionally, remaining spines were irregularly enlarged, as also occurs following *Trkb* ablation [51]. Future studies could determine whether these modifications result from aberrant vHC input and whether 7,8-DHF corrects abnormalities. Finally, while we find a robust p-ERK42/44 response to repeated 7,8-DHF injections, the ability of acute application to stimulate trkB, ERK42/44, and other factors has been questioned [52]. Identifying off-target actions of 7,8-DHF could reveal novel mechanisms by which acute application benefits animal models of depression (e.g., [46]) and Alzheimer disease (discussed in [52]).

### vHC *Trkb*.*t1* overexpression induces habits

Despite broad-spread changes in trkB:trkB.t1 ratios following subchronic CORT exposure, p-ERK42/44 was detectably reduced only in the vHC of adolescent CORT-exposed mice. These findings led us to overexpress *Trkb*.*t1* in the vHC, reducing local p-ERK42/44 and causing habit-based behavior. *Trkb*.*t1* overexpression impairs both long-term potentiation and long-term depression ([53,54], but see [55]) and reduces BDNF [56], which could potentially account for a transition away from hippocampal-dependent action selection to habit-based behaviors, which are instead associated with dorsolateral striatal and cortical sensorimotor systems [11–13,23,25].

The vHC innervates the CeA [57] and likely provides BDNF, given that the CeA expresses little *Bdnf* mRNA but abundant BDNF from non-cortical sources, and among the highest levels of amygdalar trkB [58]. Axonal BDNF transport can be trkB-dependent [59]; thus, we hypothesized that vHC *Trkb*.*t1* overexpression may render the CeA BDNF-deficient and that *Trkb*.*t1* overexpression in the CeA may similarly influence decision-making strategies. Indeed, *Trkb*.*t1* overexpression in the posterior CeA caused a deferral to habit-based strategies. This finding provides novel evidence that the healthy posterior CeA is involved in selecting behaviors according to their consequences. This may occur via regulation of appetitive arousal, rather than encoding specific action–value information per se [60]. By contrast, the anterior (and not posterior) CeA is essential to habit-based behavior, potentially due to interactions with the dorsolateral striatum [61].

Importantly, trkB.t1 is expressed in neurons [40,62,63] and glia [64–66]. Lentiviruses, as used here, preferentially infect excitatory neurons, but moderate glial infection would be anticipated [67]. Future investigations could elucidate cell-type–specific effects of *Trkb*.*t1* overexpression. Also, it is notable that we did not overexpress *Trkb*.*t1* in the mPFC. We were motivated by evidence that selective reduction of its ligand BDNF in this region fails to induce habit-based behavior [3,68]. mPFC-selective *Bdnf* knockdown does cause depression-like amotivation, however, and targeted BDNF infusions provide partial recovery from CORT-induced amotivation [3]. Thus, systemic 7,8-DHF treatment here may have ameliorated CORT-induced depression-like amotivation by acting in multiple brain regions, not strictly the vHC.

### Conclusions

To summarize, subchronic CORT exposure in adolescence imbalances trkB/trkB.t1 throughout several brain regions and selectively decreases vHC p-ERK42/44. A *Trkb*.*t1* overexpression procedure that reduces p-ERK42/44 recapitulates CORT-induced behavioral abnormalities. Interestingly, the “pro-habit” effects of vHC *Trkb*.*t1* overexpression were not age dependent, given that viral vector infusion at both P31 and P56 induced behavioral inflexibility. We argue that adolescents are not necessarily uniquely vulnerable to *Trkb*.*t1* overexpression but are rather more vulnerable to a corticosteroid-induced triggering of neurobiological factors associated with depression-like and habit-based behaviors (in this case, the concomitant elevation of trkB.t1 and reduction in p-ERK42/44 in the vHC).

## Materials and methods

### Subjects

Group-housed wildtype C57BL/6 mice (Jackson Labs) were used, except for dendritic spine imaging experiments, in which case mice were *thy1*-YFP–expressing (C57BL/6 background) [69]. Mice were provided a 12-h light cycle (0800 on) and food and water ad libitum except during instrumental conditioning when body weights of all mice were reduced to 90%–93% of baseline to motivate food-reinforced responding. Mice were males unless otherwise explicitly noted. The timing of experimental events is provided in Table 1, and timelines are also provided in the figures.

### Ethics statement

Procedures were approved by the Emory University Institutional Animal Care and Use Committee, licenses 2000973, 2002802, and 4000010, and the *Guide for the Care and Use of Laboratory Animals in Research*. In cases of euthanasia, mice were deeply anesthetized with isoflurane prior to rapid decapitation.

### CORT exposure

CORT hemisuccinate (4-pregnen-11β 21-DIOL-3 20-DIONE 21-hemisuccinate; Steraloids) was dissolved in tap water (25 μg/ml free base) according to an established protocol [3,29,30]. Mice were given CORT in place of normal drinking water. Water bottles were weighed daily, and mice were weighed every other day (Table 2). Average doses (mg/kg) of CORT were calculated by normalizing daily consumption values per cage to the total body weight of the animals in the same cage. Every 3 d, water bottles were emptied and refilled with fresh water or newly-prepared CORT solution.

Mice were exposed to CORT from P31–42 or 56–67 (in 1 cohort, P68 due to experimental error), resulting in approximately 5–9 mg/kg/d. These periods correspond to early adolescence and early adulthood in rodents [20]. Mice were euthanized at the end of the CORT exposure period, or they experienced a 2-, 4-, or 12-wk washout period as indicated.

### Forced swim stress

To compare blood serum CORT levels between CORT-exposed versus stressor-exposed mice, naive mice were exposed to forced swim stress at P31 or daily from P31–42. Mice were placed in a glass cylinder (24 cm × 15.5 cm diameter) filled to 10 cm with 22–25°C water in a dimly lit room. After 6 min, mice were allowed to dry in a warm cage lined with paper towels before being returned to the home cage. Water was changed between mice. Control mice were handled but not exposed to swim stress. Groups were also housed separately. Mice were weighed every other day (Table 2).

### Blood serum CORT

We collected trunk blood at P31 or P42. Mice were briefly anaesthetized with isoflurane and then decapitated either early in the active, dark cycle (2000 h) or late in the active cycle (0600 h). In the case of swim stress, mice were euthanized 30 min following swimming [70]. Blood was centrifuged in chilled Eppendorph tubes at 4°C for 30 min, and serum was extracted. CORT levels were analyzed in duplicate by ELISA (Assay Designs) in accordance with manufacturer’s instructions with the exception of the extraction step, which was excluded.

### Gland harvesting

Adrenal and thymus glands were extracted following euthanasia by midline dissection and weighed in pairs.

### Dendritic spine imaging, reconstruction

A widely documented consequence of repeated stressor exposure is the elimination of dendritic spines in the mPFC. As part of our efforts to test the possibility that our CORT exposure procedure recapitulated aspects of stressor exposure, brains from YFP-expressing mice were collected at the end of CORT exposure at P42 and submerged in chilled 4% paraformaldehyde for 48 h, then transferred to 30% w/v sucrose. Brains were sectioned into 40 μm-thick sections at −15°C. Dendrites on deep-layer mPFC neurons, prelimbic/medial orbital compartments, were imaged using confocal microscopy and reconstructed in 3D using Imaris software. Methods are described elsewhere [71], the only modification being that a Leica TSC SP8 microscope was used.

Eight dendrites/mouse, 16–25 μm in length and located between Bregma +1.98–+1.70, were imaged and reconstructed by a single blinded rater/experiment. In our adolescent population, dendritic spine densities were more variable than expected based on our prior investigations of prelimbic cortical neurons [3,7,35], and unblinding revealed considerable variance based on rostrocaudal positioning. Most dendrites (73%) were imaged at roughly Bregma +1.94 or +1.78. Thus, we next compared dendritic spine densities and morphological metrics by 2-factor (CORT × anatomical position) analysis of variance (ANOVA), total = 4–8 dendrites/mouse, considering each dendrite an independent sample. Values +/− 2 standard deviations from the mean were considered outliers and excluded, and the results of these comparisons are reported here.

To investigate long-term consequences of adolescent CORT exposure, dendritic spines were imaged and classified from mice exposed to CORT during adolescence, then behaviorally tested in adulthood (methods described immediately below).

### Instrumental response training

Mice were food-restricted and trained to nose poke for 20-mg grain-based food reinforcers (Bio-Serv Precision Pellets) using Med-Associates conditioning chambers equipped with 2 nose poke recesses and a food magazine. Training was initiated with a fixed ratio 1 (FR1; also called “continuous reinforcement”) schedule of reinforcement; 30 reinforcers were available for responding on each aperture (60 reinforcers/session). Sessions ended when mice acquired all reinforcers or at 70 min. 5–7 training sessions were conducted (1/d). Unless specified, response acquisition curves represent both responses/min; there were no response biases throughout.

To assess decision-making strategies, a modified version of classical instrumental contingency degradation was used, as in our prior reports (e.g., [3,35,72]). In a 25-min “non-degraded” session, 1 nose poke recess was occluded, and responding on the other was reinforced using a variable ratio 2 schedule of reinforcement. In a 25-min “degraded” session, the opposite aperture was occluded, and responding on the available aperture produced no programmed consequences. Instead, reinforcers were delivered into the magazine at a rate matched to each animal’s reinforcement rate on the previous day (that is, food pellets were delivered independently of the animal’s actions). Thus, responding on 1 aperture became significantly less likely to be reinforced than the other. The order of the sessions and which response–outcome contingency was “degraded” were counterbalanced.

The following day, a 5-min probe test was conducted in extinction. Both apertures were available. Mice that are sensitive to instrumental contingencies preferentially generate the response that is likely to be reinforced, a goal-directed response strategy; meanwhile, mice that have developed habits are insensitive to instrumental contingency degradation and generate both familiar responses equally, habitually (for further discussion of this task, see [23]).

Following test 1, responding was reinstated using an RI30-sec schedule of reinforcement for 4 d to promote the formation of stimulus–response habits [73]. 30 reinforcers were again available (60 reinforcers/session, 1 session/d). Sessions ended when mice acquired all reinforcers or at 70 min. Then, the 3-day contingency degradation and probe test protocol was repeated (“test 2”).

In a separate experiment, mice were trained to nose poke using FR1 and then RI30 schedules of reinforcement. Then, mice were tested in the contingency degradation procedure 3 times (1 session/d) to quantify the development of response inhibition.

### Context shift

To determine whether insensitivity to instrumental contingency degradation was context dependent, we utilized a “context shift” [72]: the “non-degraded” and “degraded” training sessions and probe test 2 occurred in unique chambers located in a separate room in the laboratory relative to training and test 1. The chambers were contextually distinct (containing a recessed lever and distinct odors) and configured differently (nose poke ports and house light located on different walls).

### Reinforcer devaluation

Following instrumental contingency degradation, nose poking was reinstated using an RI30 schedule of reinforcement during 3 daily training sessions. Then, prefeeding devaluation was used to assess value-based response selection. Mice were placed individually in empty shoebox-style cages for a 1-hr habituation period. Then, mice were allowed access for 30 min to either standard chow or the food pellets used during instrumental conditioning. Immediately following this prefeeding, mice were placed in the conditioning chambers, and responding in a probe test conducted in extinction was measured for 10 min. This procedure was repeated the following day with the opposite food item. Prefeeding with the reinforcer pellets, but not standard chow, reduces response rates if mice select actions based on outcome value.

Mice consumed more reinforcer pellets than chow during the prefeeding period; thus, we tested mice in a third condition in which the number of pellets available to each mouse was matched to the amount of chow that the group consumed during the prior prefeeding period. Subsequent response rates during the probe test did not differ (that is, when the pellets were restricted or not), and those following restricted access—controlling the amount of food ingested—are shown. All intake data are provided in S3 Table.

### Progressive ratio

In separate mice tested in the instrumental contingency degradation procedure, nose poke responding on 1 recess was reinstated using an FR1 schedule for 2 50-min sessions (1/d). Then, responding on a progressive ratio schedule, in which the response requirement increased by 4 with each reinforcer delivery, was measured. Sessions ended after 180 min or when mice executed no responses for 5 min. The “break point ratio” refers to the highest number of responses:reinforcers generated.

### Forced swim test

Following instrumental conditioning, mice were fed ad libitum. Within 1 wk, mice were placed in a glass cylinder (24 cm × 15.5 cm diameter) filled to 10 cm with 25°C water, as previously used to detect an increase in immobility following CORT [29]. Ten-min sessions were videotaped under dim light, and time spent immobile, defined as only movements necessary to keep the head above water, was scored by a single blinded rater. In 1 experiment, an acute stressor (19 hr water deprivation) preceded the forced swim test in half of the group; “unstressed” mice in this experiment were left undisturbed.

It is important to note that while mice in this report had ad libitum food access at the time of forced swim testing, they had all experienced modest food restriction during instrumental conditioning experiments; this could conceivably influence mobility scores (for review, [74]).

### Locomotor monitoring

Following forced swim testing, locomotor activity was monitored for 24 hr using a custom-built Med-Associates locomotor monitoring system equipped with 16 photocells. Locomotor activity was quantified in photobeam breaks across the 24-hr period, which were summed into 6-hr bins (S2 Table).

### 7,8-DHF

7,8-DHF (Sigma; dissolved in 17% dimethylsulfoxide (DMSO) and saline; 3-10mg/kg [46]) was administered i.p. daily from P39-47. This period overlapped with the end of the adolescent CORT exposure period and was determined based on pilot studies. Control mice received 17% DMSO and saline.

### Immunoblotting

Mice were euthanized at the end of the CORT exposure procedure; 12 wk following CORT (and following instrumental contingency degradation testing); or 30 min following the last of 8 daily injections of 7,8-DHF. Mice were briefly anaesthetized by isoflurane and euthanized by rapid decapitation. Brains were extracted and frozen at −80°C. Brains were sectioned into 1-mm sections using a chilled brain matrix, and the mPFC, vHC, amygdala, dorsomedial striatum, and ventral striatum were extracted using a 1-mm tissue core by a single experimenter. Tissues were homogenized by sonication in lysis buffer (200 μl: 137 mM NaCl, 20 mM tris-Hcl [pH = 8], 1% igepal, 10% glycerol, 1:100 Phosphatase Inhibitor Cocktails 2 and 3 [Sigma], 1:1,000 Protease Inhibitor Cocktail [Sigma]), and stored at −80°C. Protein concentrations were determined using a Bradford colorimetric assay (Pierce).

Equal amounts of protein were separated by SDS-PAGE on 7.5% gradient tris-glycine gels (Bio-rad). Following transfer to PVDF membrane, blots were blocked with 5% nonfat milk for 1 hr. Membranes were incubated with primary antibodies at 4°C overnight and then in horseradish peroxidase secondary antibodies for 1 hr. Immunoreactivity was assessed using a chemiluminescence substrate (Pierce) and measured using a ChemiDoc MP Imaging System (Bio-rad). Densitometry values were normalized to the control sample mean from the same membrane in order to control for fluorescence variance between gels. vHC and amygdala samples were loaded on the same gels to allow for comparisons within and between brain regions and tested at least twice.

Primary antibodies were anti-trkB (Rb, Cell Signaling, 4603s, lot 3; 1:375), anti-ERK42/44 (Rb, Cell Signaling, 9102s, lot 26; 1:2,000), anti-p-ERK42/44 (Ms, Cell Signaling, 9106s, lot 43; 1:1,000), and anti-PSD95 (Rb, Cell Signaling, 3450s, lot 2; 1:1,000). In our initial comparisons of vHC–amygdala ERK42/44, a loading control (GAPDH; Ms, Sigma, G8795, lot 044M4808V; 1:5,000) was additionally applied to further confirm equivalent loading.

### Surgery

Naïve mice were infused with a lentivirus expressing a CMV promoter and truncated trkB receptor isoform, *Trkb*.*t1*, with an HA tag (titer = 5.8 × 10^8^ iu/ml; virus described in [75,76]). Control mice were infused with lenti-GFP, also bearing a CMV promoter. Mice were anaesthetized with ketamine/xylazine and placed in a digitized stereotaxic frame (Stoelting). The scalp was incised, skin retracted, bregma and lambda identified, the head leveled, and stereotaxic coordinates corresponding to the vHC or CeA were located (−3.0 AP/−4.0 DV/±2.75 ML and −1.5 AP/−4.9 DV/±3.0 ML, respectively). Viral vectors were infused over 5 min, with 0.5 μl/side. Needles were left in place for 5 additional min prior to withdrawal and suturing. Three wk later, instrumental conditioning began. Following testing, fixed brain tissue was imaged for GFP or immunostained for HA as described [76]. Mice were infused at P31 or P56 at the same coordinates; timing did not impact behavioral outcomes.

### p-ERK42/44 immunostaining

Tissue sections from mice expressing viral vectors were blocked in a PBS solution containing 2% normal goat serum, 1% bovine serum albumin (BSA), and 0.3% Triton X-100 (Sigma) for 1 hr at room temperature. Sections were then incubated in a primary antibody solution containing 0.3% normal goat serum, 1% BSA, and 0.3% Triton X-100 at 4°C for 48 hr. p-ERK42/44 (Rb, Cell Signaling, 9102s, lot 26; 1:400) served as the primary antibody. Sections were incubated in secondary antibody solution containing 0.5% normal goat serum and 0.3% Triton X-100, with Alexa Fluor 633 (1:200; Life Technologies) serving as the secondary antibody.

Sections were imaged on a Nikon 4550s SMZ18 microscope with settings held constant. Integrated intensity (normalized to the size of the sampling area) was measured where HA or GFP staining was also detected. Sections were compared in 2 cohorts, and fluorescence values were normalized to the control mean from each respective cohort. We analyzed 1–10 sections from each mouse, with each animal contributing a single mean value to statistical analysis.

### Statistical analyses

Body weights, blood serum CORT, gland weights, response rates, response counts, break point ratios, and densitometry values were compared by 2-tailed ANOVA or *t* test using SPSS with *p* < 0.05 considered significant. Following interactions, post hoc comparisons utilized Tukey’s HSD tests, and results are indicated graphically. In mice bearing *Trkb*.*t1*-expressing viral vectors, exclusions due to mistargeted infusions and the combination of control groups resulted in considerably uneven sample sizes (reported in the captions); thus, we compared these groups using type III ANOVA. Statistical approaches to comparing dendritic spine densities and morphologies are outlined in the corresponding section above. Values lying >2 standard deviations above the means were considered outliers and excluded.

## Supporting information

S1 Data Excel file containing raw data for figures and tables. (XLSX)

S1 Fig Long-term consequences of adolescent CORT exposure on dendritic spine morphologies in the mature mPFC. Dendritic spines on excitatory neurons within the anterior mPFC of adult mice exposed to CORT during adolescence were imaged. While we detected no differences in dendritic spine densities, lengths, or head diameters, dendritic spines from CORT-exposed mice were larger in overall volume. These findings provide evidence of long-term structural effects of adolescent CORT exposure, and notably, glucocorticoid receptor blockade has differential effects, reducing dendritic spine head diameters [35]. Each symbol represents an individual dendritic spine, and groups were compared by Kolmogorov–Smirnov comparisons, * *p* = 0.03. Raw data for this figure can be found in S1 Data. CORT, corticosterone; mPFC, medial prefrontal cortex.
(TIF)

S2 Fig CORT causes habit behavior in female mice, which can be blocked by 7,8-DHF. (a) Experimental timeline. (b) Female mice exposed to CORT during adolescence acquired the instrumental responses in adulthood (*F*s < 1). Response acquisition curves represent both responses/min. (c) As in males, a history of CORT exposure biased responding towards inflexible habit-like behavior (interaction *F*_(1,7)_ = 5.6, *p* = 0.05). *n* = 4–5/group. Notably, habit behavior was detectable at an earlier time point relative to studies using males (Fig 2). This is consistent with habit biases in female mice [77]. (d) Experimental timeline. (e) A separate cohort of mice acquired the nose poke responses (*F*s ≤ 1). (f) Control mice preferentially generated the response most likely to be reinforced following instrumental contingency degradation (response *t*_14_ = 3.0, *p* = 0.009), while CORT-exposed mice failed to differentiate between the responses, responding habitually (response *t*_14_ = 0.2, *p* = 0.8). 7,8-DHF blocked these habits (response *t*_12_ = 2.7, *p* = 0.02). *n* = 7–8/group. Bars/symbols = means+SEMs, * *p* < 0.05. Raw data for this figure can be found in S1 Data. 7,8-DHF, 7,8-dihydroxyflavone; CORT, corticosterone.
(TIF)

S3 Fig Additional histological documentation associated with Fig 5. (a) Coronal brain sections from the Mouse Brain Library [27] are shown. Each trace represents the largest detected hippocampal viral vector spread in a given mouse. (b) Separate mice received CeA infusions. The center of each *Trkb*.*t1*-expressing viral vector spread is indicated. The largest and smallest infusion sites are documented in Fig 5.CeA, central nucleus of the amygdala; trkB, tyrosine kinase receptor B; trkB.t1, truncated trkB.
(TIF)

S1 Table Gland weights in mice with a history of CORT±7,8-DHF. When mice were euthanized in adulthood following a history of CORT±7,8-DHF treatment (Fig 4), adrenal and thymus gland weights did not differ (all *p* > 0.05). Values indicate gland weights as a percentage of total body weight. 7,8-DHF dosing (in mg/kg) is indicated in parentheses. Raw data for this table can be found in S1 Data. 7,8-DHF, 7,8-dihydroxyflavone; CORT, corticosterone.
(DOCX)

S2 Table Locomotor monitoring of mice exposed to CORT during adolescence ± 7,8-DHF. The locomotor activity of adult mice exposed to CORT±7,8-DHF (3 mg/kg) as adolescents was monitored over 24 hr following the forced swim test (Fig 4). There were no effects of CORT, 7,8-DHF, CORT × 7,8-DHF interactions, or any interactions with time (main effect and interaction *p* > 0.05). Units of measure are photobeam breaks, and these findings are consistent with no locomotor effects of repeated 7,8-DHF treatment in rats [78]. Raw data for this table can be found in S1 Data. 7,8-DHF, 7,8-dihydroxyflavone; CORT, corticosterone.
(DOCX)

S3 Table Food intake associated with reinforcer devaluation experiments in Fig 2. Male mice with a history of CORT exposure during adolescence or adulthood consumed more of the reinforcer pellets (“devalued”) than regular chow (“non-devalued”) when given free access to each before a probe test conducted in extinction. Thus, in a third condition, the number of pellets available during the prefeeding period was matched to the amount of chow that the group consumed previously. Intake values are reported in grams. Raw data for this table can be found in S1 Data. CORT, corticosterone.
(DOCX)

## Abbreviations

7,8-DHF: 7,8-dihydroxyflavone
BDNF: Brain-derived Neurotrophic Factor
BSA: bovine serum albumin
CA: Cornu amonis
CeA: central nucleus of the amygdala
CORT: corticosterone
DMSO: dimethylsulfoxide
ERK42/44: extracellular signal-regulated kinase 42/44
FR1: fixed ratio 1
GFP: green fluorescent protein
i.p.: intraperitoneal
mPFC: medial prefrontal cortex
P: postnatal day
p-ERK: phosphorylated ERK
PFC: prefrontal cortex
PSD95: postsynaptic density 95
RI: random interval
trkB: tyrosine kinase receptor B
trkB.t1: truncated trkB
vHC: ventral hippocampus
YFP: yellow fluorescent protein
