# Supplementary figures and images for: Regulation of actions and habits by ventral hippocampal trkB and adolescent corticosteroid exposure
Source: PLoS Biol. 2017 Nov 29;15(11):e2003000. doi: 10.1371/journal.pbio.2003000 (PMC5724896; doi:10.1371/journal.pbio.2003000)

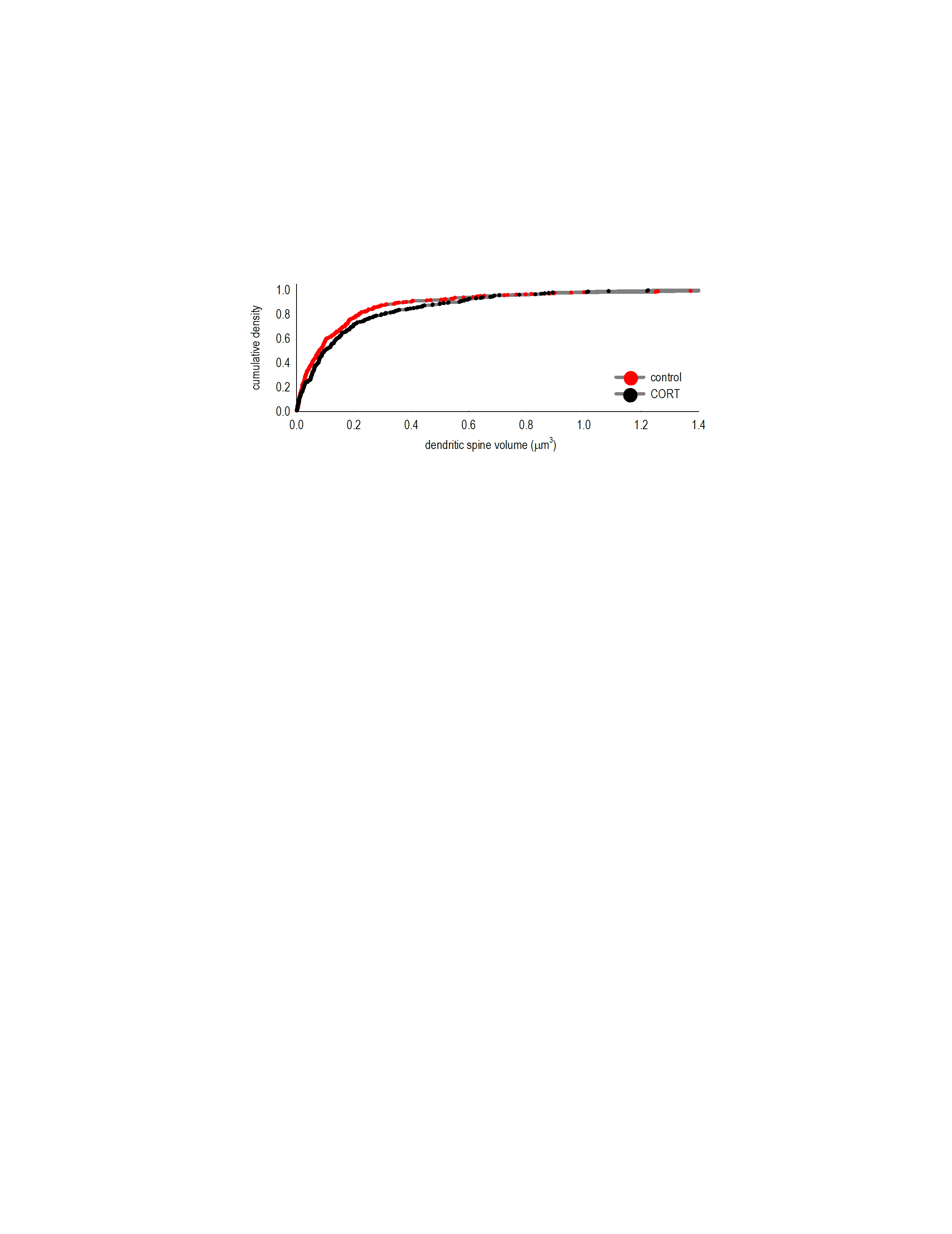

Supplement: S1 Fig — Dendritic spines on excitatory neurons within the anterior mPFC of adult mice exposed to CORT during adolescence were imaged. While we detected no differences in dendritic spine densities, lengths, or head diameters, dendritic spines from CORT-exposed mice were larger in overall volume. These findings provide evidence of long-term structural effects of adolescent CORT exposure, and notably, glucocorticoid receptor blockade has differential effects, reducing dendritic spine head diameters [35]. Each symbol represents an individual dendritic spine, and groups were compared by Kolmogorov–Smirnov comparisons, * p = 0.03. Raw data for this figure can be found in S1 Data. CORT, corticosterone; mPFC, medial prefrontal cortex. (TIF) [file pbio.2003000.s002.TIF]

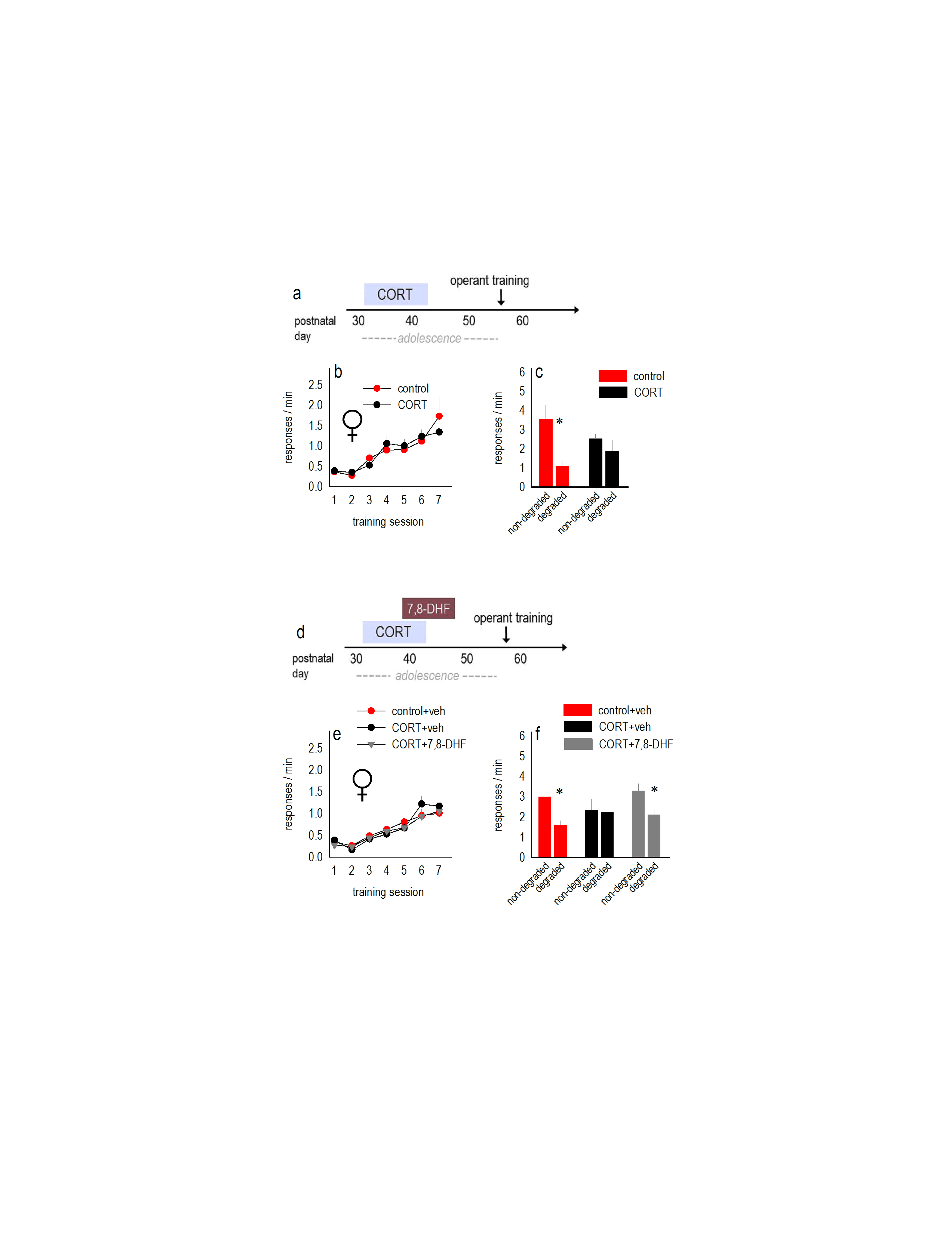

Supplement: S2 Fig — (a) Experimental timeline. (b) Female mice exposed to CORT during adolescence acquired the instrumental responses in adulthood (Fs < 1). Response acquisition curves represent both responses/min. (c) As in males, a history of CORT exposure biased responding towards inflexible habit-like behavior (interaction F(1,7) = 5.6, p = 0.05). n = 4–5/group. Notably, habit behavior was detectable at an earlier time point relative to studies using males (Fig 2). This is consistent with habit biases in female mice [77]. (d) Experimental timeline. (e) A separate cohort of mice acquired the nose poke responses (Fs ≤ 1). (f) Control mice preferentially generated the response most likely to be reinforced following instrumental contingency degradation (response t14 = 3.0, p = 0.009), while CORT-exposed mice failed to differentiate between the responses, responding habitually (response t14 = 0.2, p = 0.8). 7,8-DHF blocked these habits (response t12 = 2.7, p = 0.02). n = 7–8/group. Bars/symbols = means+SEMs, * p < 0.05. Raw data for this figure can be found in S1 Data. 7,8-DHF, 7,8-dihydroxyflavone; CORT, corticosterone. (TIF) [file pbio.2003000.s003.TIF]

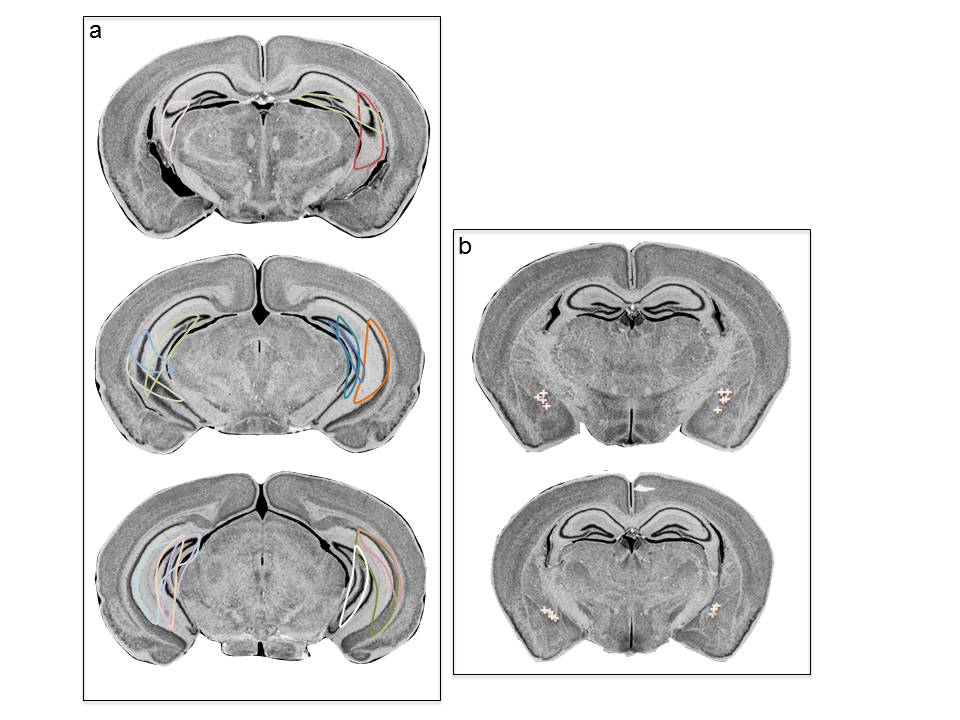

Supplement: S3 Fig — (a) Coronal brain sections from the Mouse Brain Library [27] are shown. Each trace represents the largest detected hippocampal viral vector spread in a given mouse. (b) Separate mice received CeA infusions. The center of each Trkb.t1-expressing viral vector spread is indicated. The largest and smallest infusion sites are documented in Fig 5.CeA, central nucleus of the amygdala; trkB, tyrosine kinase receptor B; trkB.t1, truncated trkB. (TIF) [file pbio.2003000.s004.tif]

|  | |  |  |  |  |
| --- | --- | --- | --- | --- | --- |
|  | |  | adrenal |  | thymus |
|  | |  | Mean ± SEM |  | Mean ± SEM |
|  | |  |  |  |  |
| 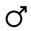*Adolescence*  *(7,8-DHF)* | control | veh | 0.020 ± 0.0011 |  | 0.23 ± 0.020 |
| DHF(3) | 0.017 ± 0.0014 |  | 0.23 ± 0.014 |
| DHF(10) | 0.016 ± 0.0025 |  | 0.22 ± 0.011 |
| CORT | veh | 0.020 ± 0.0017 |  | 0.23 ± 0.019 |
| DHF(3) | 0.016 ± 0.0012 |  | 0.24 ± 0.012 |
| DHF(10) | 0.019 ± 0.0013 |  | 0.22 ± 0.028 |
|  |  |  |  |  |  |

Supplement: S1 Table — When mice were euthanized in adulthood following a history of CORT±7,8-DHF treatment (Fig 4), adrenal and thymus gland weights did not differ (all p > 0.05). Values indicate gland weights as a percentage of total body weight. 7,8-DHF dosing (in mg/kg) is indicated in parentheses. Raw data for this table can be found in S1 Data. 7,8-DHF, 7,8-dihydroxyflavone; CORT, corticosterone. (DOCX) [file pbio.2003000.s005.docx]
