# Supplementary material for: Regulation of actions and habits by ventral hippocampal trkB and adolescent corticosteroid exposure
Source: PLoS Biol. 2017 Nov 29;15(11):e2003000. doi: 10.1371/journal.pbio.2003000 (PMC5724896; doi:10.1371/journal.pbio.2003000)
Supplement: S2 Table — The locomotor activity of adult mice exposed to CORT±7,8-DHF (3 mg/kg) as adolescents was monitored over 24 hr following the forced swim test (Fig 4). There were no effects of CORT, 7,8-DHF, CORT × 7,8-DHF interactions, or any interactions with time (main effect and interaction p > 0.05). Units of measure are photobeam breaks, and these findings are consistent with no locomotor effects of repeated 7,8-DHF treatment in rats [78]. Raw data for this table can be found in S1 Data. 7,8-DHF, 7,8-dihydroxyflavone; CORT, corticosterone. (DOCX) [file pbio.2003000.s006.docx]

|  | |  |  |  |  |  |  |  |  |
| --- | --- | --- | --- | --- | --- | --- | --- | --- | --- |
|  | | hours | 1-6 |  | 7-12 |  | 13-18 |  | 19-24 |
|  | |  | Mean ± SEM |  | Mean ± SEM |  | Mean ± SEM |  | Mean ± SEM |
|  | |  |  |  |  |  |  |  |  |
| *Adolescence*  *(7,8-DHF)* | control | veh | 55275 ± 3441 |  | 46079 ± 7863 |  | 29327 ± 10458 |  | 27549 ± 4993 |
|  |  | DHF | 37538 ± 2804 |  | 32389 ± 3672 |  | 26233 ± 11994 |  | 32700 ± 10015 |
|  | CORT | veh | 43568 ± 5628 |  | 42631 ± 7505 |  | 18455 ± 4943 |  | 19427 ± 3534 |
|  |  | DHF | 49734 ± 6421 |  | 34546 ± 4323 |  | 29135 ± 8478 |  | 39562 ± 14695 |
|  |  |  |  |  |  |  |  |  |  |
