# Supplementary material for: Regulation of actions and habits by ventral hippocampal trkB and adolescent corticosteroid exposure
Source: PLoS Biol. 2017 Nov 29;15(11):e2003000. doi: 10.1371/journal.pbio.2003000 (PMC5724896; doi:10.1371/journal.pbio.2003000)
Supplement: S3 Table — Male mice with a history of CORT exposure during adolescence or adulthood consumed more of the reinforcer pellets (“devalued”) than regular chow (“non-devalued”) when given free access to each before a probe test conducted in extinction. Thus, in a third condition, the number of pellets available during the prefeeding period was matched to the amount of chow that the group consumed previously. Intake values are reported in grams. Raw data for this table can be found in S1 Data. CORT, corticosterone. (DOCX) [file pbio.2003000.s007.docx]

|  |  |  |  |  |  |  |
| --- | --- | --- | --- | --- | --- | --- |
|  |  | chow |  | pellet |  | matched pellet |
|  |  | Mean ± SEM |  | Mean ± SEM |  | Mean ± SEM |
|  |  |  |  |  |  |  |
| *Adolescence* | control | 0.38 ± 0.032 |  | 0.74 ± 0.09 |  | 0.40 ± 0.00 |
|  | CORT | 0.41 ± 0.048 |  | 0.69 ± 0.12 |  | 0.40 ± 0.00 |
| *Adulthood* | control | 0.53 ± 0.052 |  | 1.01 ± 0.13 |  | 0.50 ± 0.00 |
|  | CORT | 0.50 ± 0.044 |  | 0.97 ± 0.15 |  | 0.50 ± 0.00 |
|  |  |  |  |  |  |  |
